# Differential distribution of the sodium‐activated potassium channels slick and slack in mouse brain

**DOI:** 10.1002/cne.23934

**Published:** 2015-12-15

**Authors:** Sandra Rizzi, Hans‐Günther Knaus, Christoph Schwarzer

**Affiliations:** ^1^ Division of Molecular and Cellular PharmacologyMedical University of Innsbruck6020InnsbruckAustria; ^2^ Department of PharmacologyMedical University of Innsbruck6020InnsbruckAustria

**Keywords:** Slo2 channels, KCNT1, KCNT2, mouse brain, expression, distribution

## Abstract

The sodium‐activated potassium channels Slick (Slo2.1, KCNT2) and Slack (Slo2.2, KCNT1) are high‐conductance potassium channels of the Slo family. In neurons, Slick and Slack channels are involved in the generation of slow afterhyperpolarization, in the regulation of firing patterns, and in setting and stabilizing the resting membrane potential. The distribution and subcellular localization of Slick and Slack channels in the mouse brain have not yet been established in detail. The present study addresses this issue through *in situ* hybridization and immunohistochemistry. Both channels were widely distributed and exhibited distinct distribution patterns. However, in some brain regions, their expression overlapped. Intense Slick channel immunoreactivity was observed in processes, varicosities, and neuronal cell bodies of the olfactory bulb, granular zones of cortical regions, hippocampus, amygdala, lateral septal nuclei, certain hypothalamic and midbrain nuclei, and several regions of the brainstem. The Slack channel showed primarily a diffuse immunostaining pattern, and labeling of cell somata and processes was observed only occasionally. The highest Slack channel expression was detected in the olfactory bulb, lateral septal nuclei, basal ganglia, and distinct areas of the midbrain, brainstem, and cerebellar cortex. In addition, comparing our data obtained from mouse brain with a previously published study on rat brain revealed some differences in the expression and distribution of Slick and Slack channels in these species. J. Comp. Neurol. 524:2093–2116, 2016. © 2015 The Authors The Journal of Comparative Neurology Published by Wiley Periodicals, Inc.

**Abbreviations**
3: oculomotor nucleus
7: facial nucleus
aca: anterior commissure
AcbC: nucleus accumbens core
AcbSh: nucleus accumbens shell
aci: intrabulbar anterior commissure
acp: anterior commissure posterior
AInt: anterior interposed nucleus
Amb: nucleus ambiguous
Amy: amygdaloid nuclei
AOB: accessory olfactory bulb
AON: anterior olfactory area
APT: anterior pretectal nuclei
Arc: arcuate nucleus
ATg: anterior tegmental nucleus
BLA: basolateral amygdaloid nucleus
BMP: basomedial amygdaloid nucleus
BST: bed nucleus of stria terminalis
BSTIA: intra‐amygdalar part of bed nuclei stria terminalis
cc: corpus callosum
Ce: central amygdaloid nucleus
CL: central lateral thalamic nucleus
CM: centromedial thalamic nucleus
CPu: caudate putamen
DCN: deep cerebellar nuclei
df: dorsal fornix
DLG: dorsal lateral geniculate nucleus
dlo: dorsolateral olfactory tract
DM: dorsomedial hypothalamic nucleus
Ect: ectorhinal cortex
EPl: external plexiform layer of the main olfactory bulb
EPlA: external plexiform layer of the accessory olfactory bulb
EW: Edinger‐Westphal nucleus
f: fornix
fr: fasciculus retroflexus
Gi: gigantocellular nucleus
Gl: glomerular layer of the main olfactory bulb
GlA: glomerular layer of the accessory olfactory bulb
GraCb: granule cell layer of the cerebellar cortex
GrDG: granule cell layer of dentate gyrus
GrO: granule layer
Hb: habenular nuclei
Hip: hippocampus
ic: internal capsule
ICj: islands of Calleja
IMD: intermediodorsal thalamic nucleus
Int: interposed nucleus
IOD: dorsal accessory nucleus of inferior olive
IODM: dorsomedial cell group of inferior olive
IOM: medial nucleus of inferior olive
IOPr: principal nucleus of inferior olive
IPC: caudal part interpeduncular nucleus
IPI: intermedial subnucleus interpeduncular nucleus
IPl: internal plexiform layer
IPL: lateral subnucleus interpeduncular nucleus
IPN: interpeduncular nucleus
La: lateral amydaloid nucleus
Lat: lateral (dentate) cerebellar nucleus
LatPC: parvocellular part of lateral cerebellar nucleus
LGP: lateral globus pallidus
LHb: lateral habenula
LMol: stratum lacunosum moleculare hippocampi
lo: lateral olfactory tract
LSD: dorsal part of lateral septal nucleus
LSI: intermediate part of septal nucleus
LSO: lateral superior olive
LSV: ventral part of lateral septal nucleus
M1: primary motor cortex
M2: secondary motor cortex
Med: medial (fastigal) cerebellar nucleus
MGP: medial globus pallidus
MHb: medial habenula
Mi: mitral layer
MiA: mitral layer of the accessory olfactory bulb
ml: medial lemniscus
mlf: medial longitudinal fasciculus
MnPO: median preoptic nucleus
MnR: median raphe nucleus
Mol: stratum moleculare of dentate gyrus
MolCb: molecular layer cerebellar cortex
MPA: medial preoptic area
ns: nigrostriatal bundle
PAG: periaqueductal gray
PC: paracentral thalamic nucleus
pc: posterior commissure
Pint: posterior interposed nucleus
Pir: piriform cortex
PMR: paramedian raphe nucleus
Pn: pontine nucleus
PnC: caudal part of pontine nucleus
PnO: oral part pontine nucleus
PnR: pontine raphe nucleus
PoDG: polymorph layer of dentate gyrus
Pur: Purkinje cell layer cerebellar cortex
PV: paraventricular thalamic nucleus
PVA: anterior paraventricular thalamic nucleus
Py: stratum pyramidale hippocampi
Rad: stratum radiatum hippocampi
Rbd: rhabdoid nucleus
RLi: rostral linear nucleus raphe
RMC: magnocelluar part of red nucleus
RMg: raphe magnus nucleus
RSA: agranular zone of retrosplenial cortex
RSG: granular zone of retrosplenial cortex
Rt: reticular thalamic nucleus
RtTg: reticulotegmental nucleus pons
S1BF: barrel field of primary somatosensory cortex
S1Tr: trunk region of primary somatosensory cortex
S2: secondary somatosensory cortex
SFi: septofimbrial nucleus
SFO: subfornical organ
SLu: stratum lucidum hippocampi
SNC: substantia nigra pars compacta
SNR: substantia nigra pars reticulata
SuG: superior colliculus superficial gray
TS: triangular septal nucleus
Tz: trapezoid body
VeCb: vestibulocerebellar nucleus
VMH: ventromedial thalamic nuclei
VMPO: ventromedial preoptic nucleus
VP: ventral pallidum
VPT: ventral posterior thalamic nuclei
VTA: ventral tegmental area
wm: white matter
xscp: decussatio superior cerebellar peduncule

More than 30 years ago, the existence of native sodium‐activated potassium currents was demonstrated through electrophysiological experiments in guinea pig cardiomyocytes (Kameyama et al., 1984). Since then, the presence of native sodium‐activated potassium channels has been reported in diverse myocytes (Kim et al., 2007; Re et al., 1990), pancreatic duct epithelial cells (Hayashi and Novak, 2013), thick ascending limb of Henle's loop in the kidney (Paulais et al., 2006), various mammalian neurons (Egan et al., 1992a, 1992b; Kaczmarek, 2013), and *Xenopus* oocytes (Egan et al., 1992a). At present, two distinct ion channels giving rise to an outward rectifying potassium current activated upon rises in internal sodium ions have been described (Bhattacharjee and Kaczmarek, 2005; Salkoff et al., 2006; Yuan et al., 2003). The sodium‐activated potassium channels Slick (sequence like an intermediate potassium channel, Slo2.1) and Slack (sequence like a calcium‐activated potassium channel, Slo2.2) belong to the structurally related high‐conductance potassium channels of the Slo family. Slick and Slack channels are composed of pore‐forming alpha subunits probably assembling into tetrameric channels (Bhattacharjee and Kaczmarek, 2005; Salkoff et al., 2006). Recent studies suggest that the alpha subunits of Slick and Slack channels are forming heteromeric channels with distinct biophysical properties *in vitro*. Moreover, immunoprecipitation studies performed on rat olfactory bulb and brainstem provided the first evidence that Slick and Slack channels might assemble into protein complexes *in vivo* (Chen et al., 2009). Channel diversity may be further increased by the presence of different isoforms. Although for the Slick channel no splice isoforms have been described, five different Slack channel isoforms differing in their N‐terminal region have been identified so far (Brown et al., 2008).

Native sodium‐dependent potassium currents have been reported to vary in their unitary conductance, subconductance states, channel open probabilities, sensitivity to sodium ions and rundown in excised patches (Bhattacharjee and Kaczmarek, 2005; Dryer, 1994; Egan et al., 1992a; Kaczmarek, 2013). Native sodium‐activated potassium channels may be activated upon sodium‐influx possibly mediated by a variety of sodium‐gating ion channels, such as voltage‐gated sodium channels and ionotropic ligand‐gated receptors (AMPA and NMDA glutamate receptors), as well as nonselective voltage‐gated cation channels (Kaczmarek, 2013). In contrast to previous ideas that sodium‐activated potassium channels are activated only under sustained intracellular sodium accumulation, recent studies suggest that a single action potential and, moreover, a persistent, noninactivating inward sodium current are sufficient to activate sodium‐dependent potassium currents (Budelli et al., 2009; Gao et al., 2008; Hage and Salkoff, 2012; Liu and Stan Leung, 2004; Wallen et al., 2007). For neurons, sodium‐activated potassium channels were shown to be involved in adapting the firing pattern of neurons, in the generation of the slow afterhyperpolarization (sAHP) and depolarizing afterpotential (DAP), and in stabilization and setting of the resting membrane potential (Franceschetti et al., 2003; Gao et al., 2008; Liu and Stan Leung, 2004; Sanchez‐Vives et al., 2000; Yang et al., 2007; Zhang et al., 2010). Mutations in the Slack gene have been associated with diverse types of seizures in humans (Barcia et al., 2012; Heron et al., 2012; Ishii et al., 2013; Martin et al., 2014).

Several electrophysiological experiments (Berg et al., 2007; Budelli et al., 2009; Egan et al., 1992a; Franceschetti et al., 2003; Kim and McCormick, 1998; Sanchez‐Vives et al., 2000; Schwindt et al., 1989; Yang et al., 2007; Zamalloa et al., 2009; Zhang et al., 2010) as well as studies establishing the distribution pattern of Slick and Slack channels in the rat brain (Bhattacharjee et al., 2002, 2005) suggest a widespread expression of the channels in mammalian brain. Currently, no specific activators or inhibitors for the Slick and Slack channels are available, hampering discrimination between native Slick and Slack channels in electrophysiological recordings. Consequently, detailed knowledge of the distinct distribution patterns of Slick and Slack channels is of particular interest. However, the expression pattern of Slick and Slack channels in mouse brain is still widely unexplored, even though the mouse is one of the most commonly used species in biomedical research.

The present study investigates the distribution of Slick and Slack channels in mouse brain. To address this issue, we performed *in situ* hybridization and immunohistochemistry. Our data suggest widespread but distinct distributions of both channels in the mouse brain. In addition, we compared our data with previously published data obtained from rat brain and observed some differences in the expression and distribution of Slick and Slack channels in mouse vs. rat brain.

## MATERIALS AND METHODS

### Animals

C57BL/6J mice were housed and handled in accordance with the guidelines of Austrian law, which are in line with the directive of the European Union (2010/63/EU) for the use of laboratory animals. All procedures involving animals were approved by the Austrian Animal Experimentation Ethics Board in compliance with the European Convention for the Protection of Vertebrate Animals Used for Experimental and Other Scientific Purposes. Every effort was taken to minimize the number and suffering of animals used. Mice were kept in type IIL single ventilated cages, in groups of up to four mice of the same sex. Mice had free access to food and water and were kept under controlled conditions (temperature 23°C, relative humidity ∼45%) with lights on (50–60 lux) at 6:30 am and lights off at 6:30 pm. The cages were bedded with wooden chips and had a plastic tube as a hiding place.

### Transfection of HEK cells

HEK tsA‐201 (human embryonic kidney) cells were grown in high‐glucose Dulbecco's modified Eagle's medium (DMEM; Sigma‐Aldrich) supplemented with 10% (v/v) fetal bovine serum (Gibco, Invitrogen) and 10 U/ml penicillin/streptomycin (PAA) and maintained at 37°C and 5% CO_2_. Cells were transfected with either pcDNA3‐Slo2.1 (Slick alpha subunit) plasmid (a kind gift of Heather McClafferty) or pcDNA3‐Slo2.2 (Slack alpha subunit) plasmid (a kind gift of Larry Salkoff, addgene plasmid 16214) using Metafectene Pro (Biontex), following the manufacturer's guidelines. Transfected cells were harvested and lysed in a buffer containing 10 mM Tris‐HCl, 1 mM EDTA, 1 mM phenylmethylsulfonylfluoride (PMSF; Sigma‐Aldrich) supplemented with protease inhibitor cocktail (Complete tablets, Roche). After trituration and low‐speed centrifugation steps, lysed cells were centrifuged at 150,000*g* for 30 minutes and resuspended in 20 mM Tris‐HCl, pH 7.4. Yielded cell membranes were snap‐frozen in liquid nitrogen and stored at –80°C until use.

### Preparation of crude plasma membranes (P2 pellet)

For the preparation of crude plasma membranes (P2 pellet), two adult (3‐4 months old) C57BL/6J mice were killed by cervical dislocation, and brains were quickly removed. Mouse brains were dissected, and tissues were placed into ice‐cold homogenization buffer (320 mM sucrose, 10 mM Tris‐HCl, pH 7.4, 1 mM EDTA, 0.5 mM PMSF supplemented with protease inhibitors [Complete tablets]). Homogenized brain tissue was centrifuged at 1,080*g* for 10 minutes at 4°C. Supernatant was collected and spun at 200,000*g* for 30 minutes at 4°C. The pellet was subsequently resuspended in 20 mM Tris‐HCl, pH 7.4, and the previous centrifugation step was repeated (washing step). The pellet was resuspended in 20 mM Tris‐HCl, pH 7.4, snap‐frozen in liquid nitrogen, and stored at –80°C.

### Western blots

Western blots were performed as described elsewhere (Sailer et al., 2004). In brief, 10 μg of either untransfected or HEK cell lysates transfected with either pcDNA3‐Slo2.1 or pcDNA3‐Slo2.2 or 20 μg of purified synaptic plasma membrane vesicles was separated by 4–15% precast TGX gel (Bio‐Rad, Hercules, CA) for SDS‐PAGE and transferred onto PVDF membranes (Millipore, Bedford, MA). After blocking in PBS containing 0.05% Tween‐20 (Roth) and 3% bovine serum albumin (Roth) for 1 hour, membranes were incubated with monoclonal mouse anti‐Slick (1:1,000, clone N11/33; NeuroMab) or anti‐Slack (1:3,000, clone N3/26; NeuroMab) antibody for 2.5 hours at room temperature. Membranes were incubated with HRP‐labeled goat anti‐mouse IgG1 (1:150,000, A10551; Life Technologies) for 40 minutes. Western blots were developed with chemiluminescent HRP substrate (Millipore), and PVDF membranes were exposed to Amersham hyperfilm.

### Tissue preparation

Three‐ to four‐month‐old C57BL/6J mice were deeply anesthetized with thiopental (150 mg/kg; Sandoz) and perfused via the left cardiac ventricle with phosphate‐buffered saline (PBS), pH 7.4, for 2 minutes, followed by 4% ice‐cold paraformaldehyde (PFA; Merck) in PBS for 10 minutes. Finally, mice were perfused with PBS for 2 minutes to remove remaining PFA. Brains were quickly removed and transferred into 20% sucrose (Roth) in PBS containing 0.05% (w/v) sodium azide (Merck‐Millipore) as preservative. Brains were snap‐frozen in –50°C 2‐methylbutan (Roth) and sliced with a cryostat. Alternatively, after perfusion, brains were transferred into 8% sucrose in PBS containing 0.05% (w/v) sodium azide and sliced with a vibratome (Leica). Forty‐micrometer free‐floating coronal brain sections were used for immunohistochemistry.

### Immunohistochemistry

Brain sections were blocked in blocking buffer (50 mM Tris‐HCl, 150 mM NaCl, 0.2% Triton X‐100 [Roth], 10% normal goat serum [PAA], 0.1% sodium azide) for 1.5 hours at room temperature. Thereafter, brain slices were incubated overnight at room temperature in blocking buffer containing the primary antibody: anti‐Slick antibody 1:500 (clone N11/33, IgG1 subtype; NeuroMab) or anti‐Slack antibody 1:400 (clone N3/26, IgG1 subtype; NeuroMab). After being washed three times for 10 minutes each in washing buffer (50 mM Tris‐HCl, 150 mM NaCl, 0.2% Triton X‐100), brain slices were incubated in washing buffer containing HRP‐labeled goat anti‐mouse IgG1 (1:500, A10551; Life Technologies) secondary antibody. Slices were washed in washing buffer, and immunoreaction products were visualized by incubation in 3,3′‐diaminobenzidine (0.5 mg/ml; Sigma‐Aldrich) and hydrogen peroxide (0.05%; Roth) for 2.5–10 minutes. Immunohistochemical experiments with nonimmune IgG1 antibody (MABC002; Chemicon, Millipore) or experiments in which the primary antibody was omitted served as negative controls. Images were taken with a Zeiss Axioplan 2 microscope, and image acquisition was performed in AxioVision rel 4.8.

**Table 1:** Antibodies Used

Antigen	Description of immunogen	Source, host species, catalog No., clone No., RRID	Concentration used (ng/μl)
Anti‐KCNT1/Slo2.2/Slack sodium‐activated potassium channel	Fusion protein amino acids 1168–1237 (cytoplasmic C‐terminus) of rat Slo2.2	UCDavis/NIH NeuroMab Facility, mouse monoclonal IgG1, clone N3/26, 75‐051, AB_2131855	WB: 0.3 IHC: 2.5
Anti‐KCNT2/Slo2.1/Slick sodium‐ and chloride‐activated ATPsensitive potassium channel	Fusion protein amino acids 564–624 (cytoplasmic C‐terminus) of mouse Slo2.1	UCDavis/NIH NeuroMab Facility, mouse monoclonal IgG1, clone N11/33, 75‐055, AB_2296599	WB: 0.1 IHC: 0.2

### Characterization of antibodies

**Figure 1:**
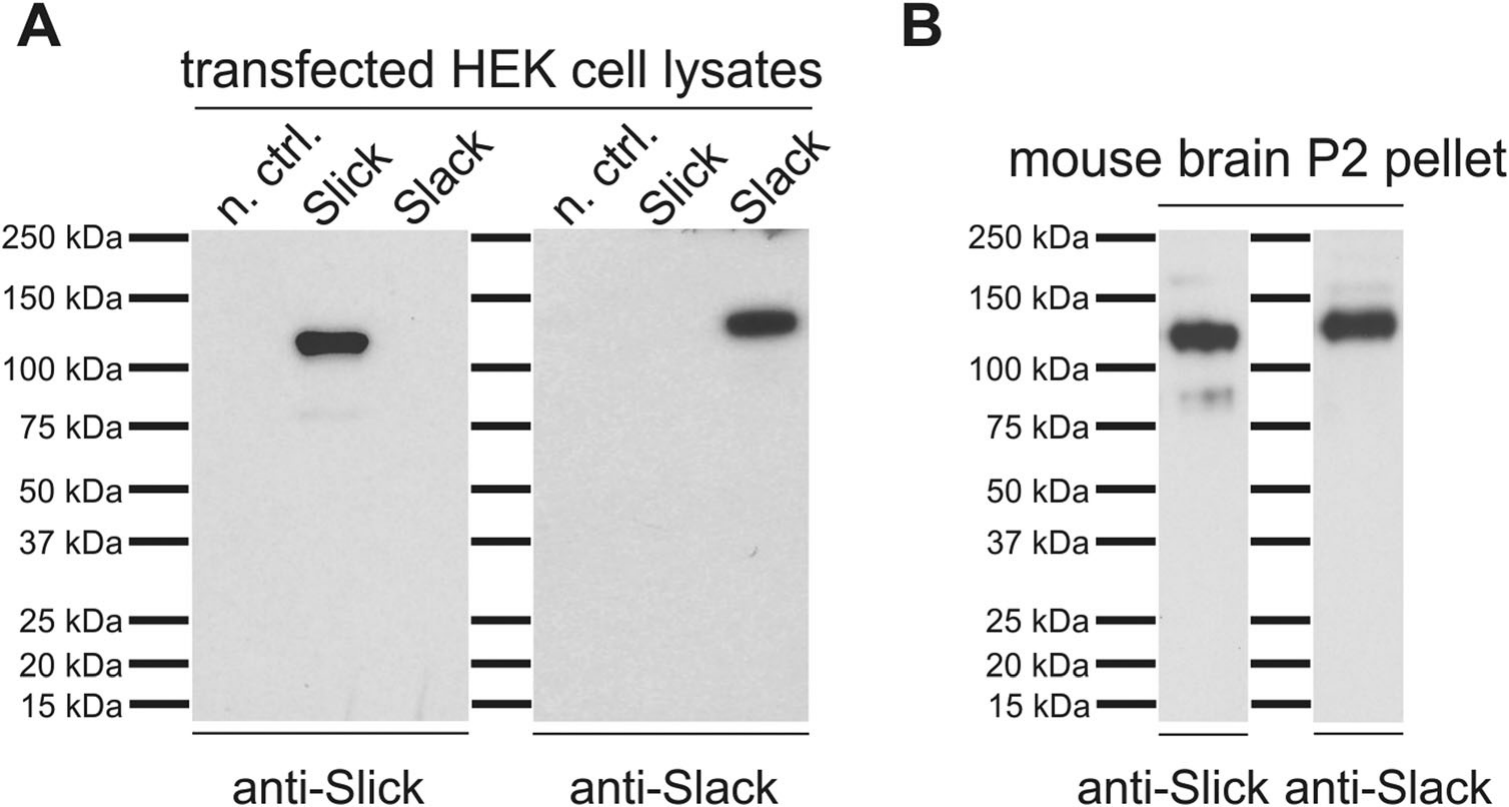
Characterization of Slick and Slack channel antibodies in Western blot analysis. Western blots were conducted using either transfected HEK‐293 cell lysates (**A**) or crude plasma membranes (P2 pellet) derived from WT mouse brains (**B**). Untransfected cells were used as negative control (n. ctrl.). Proteins were separated by 4–15% SDS‐PAGE and transferred onto PVDF membranes. Incubation with mouse monoclonal antibodies directed against C‐termini of Slick and Slack channels labeled single bands for Slick and Slack protein (predicted molecular weight for Slick 130 kDa and for Slack 138 kDa) in Western blots. Note that the Slick channel‐specific antibody does not show any cross‐reactivity to the Slack channel and vice versa.

Slick and Slack channels share 74% amino acid sequence identity, with most divergence within their N‐terminal regions. Although for the Slick channel no isoforms have been described, five different Slack channel isoforms have been identified so far. The Slack channel isoforms differ only in their N‐terminal region (Brown et al., 2008). Commercially available mouse monoclonal C‐terminal anti‐Slick and anti‐Slack (pan‐Slack) channel antibodies were used for immunohistochemical experiments. Specificity of the antibodies was assessed by Western blot analysis. Western blots were performed with HEK‐293 cell lysates transfected with plasmids encoding the cDNA sequences of Slick and Slack channels; untransfected HEK‐293 cells served as negative control. Staining with the anti‐Slick antibody resulted in a distinct band of a molecular weight of approximately 130 kDa whereas the anti‐Slack antibody labeled a single band at a molecular weight of approximately 138 kDa (Fig. 1A). Because the sodium‐activated Slick and Slack channels are structurally highly related, we also tested for cross‐reactivity on transfected HEK‐293 cell lysates. As shown in Figure 1A, the anti‐Slick channel antibody did not show any cross‐reactivity to the Slack channel protein and vice versa. In addition, we performed Western blot analysis with crude mouse brain membranes (P2 pellet). Both antibodies showed distinct bands of the predicted molecular weight (Fig. 1B).

Immunohistochemical experiments with a nonimmune IgG of the same subtype or experiments in which the primary antibody was omitted were run in parallel (shown in Fig. 3F,H, respectively). Our control experiments resulted in very faint background staining, indicating that both antibodies used are specifically recognizing Slick and Slack channel protein.

### *In situ* hybridization

Three‐ to four‐month‐old C57BL/6J mice were killed by cervical dislocation, and brains were quickly removed. Brains were snap‐frozen in –50°C 2‐methylbutane and subsequently sliced into 20‐μm sections with a cryostat. We performed *in situ* hybridization as described in detail elsewhere (Wittmann et al., 2009). In brief, four different ^35^S‐labeled single‐stranded 45mer antisense DNA‐oligonucleotides complementary to the Slick channel mRNA (5′‐AACGACAAAGAGCTTGGAGGACCATGTTTCAGGAGTGACATCCCC‐3′ starting from position 736, 5′‐ATCTGTAGTACTCTCCGAACTTGCACATCCATCTCCGTGGGACAC‐3′ starting from position 1005, 5′‐TAACATACTGATGCTGAACACTCTTCCCGCAGCAAAAGGCAGTCG‐3′ starting from position 2635, 5′‐TTCATAGAACAAAGAAACCCCGATCCTGGTATGGTGTCCAGTCCC‐3′ starting from position 2742) or Slack channel mRNA (5′‐CTGCAAGGCCCCGTCTCTTCTGCTTCTCCTCCTGTTTGAAGATG‐3′ starting from position 1868 of splice variant 1, also contained in splice variants 2 and 3) were used. Sense probes of Slick and Slack channel mRNA were used as specificity controls. Coronal 20‐μm mouse brain sections were incubated with radiolabeled oligonucleotides for approximately 18 hours at 52°C. Brain slices were washed for 4 × 15 minutes in 1× SSC at 62°C, dried, and exposed to MR film (Kodak Biomax) for 8–14 days. Sections were dipped into radiation‐sensitive emulsion (Kodak NTB; Integra Biosciences) and exposed for 3–4 weeks. Dipped sections were faintly Nissl counterstained (Franklin and Paxinos, 1997).

### Data analysis

Labeled sections were analyzed with a Zeiss Axioplan 2 microscope. Intensity of immunoreactivity and *in situ* hybridization labeling were evaluated with a qualitative scale of –, absent/background levels; +, weak; ++, moderate; +++, strong; ++++, very strong. Anatomical terminology and neuroanatomical definitions of brain areas were based on the Franklin and Paxinos mouse brain atlas (Franklin and Paxinos, 1997).

## RESULTS

### General distribution of Slick and Slack channels in the mouse brain

**Figure 2:**
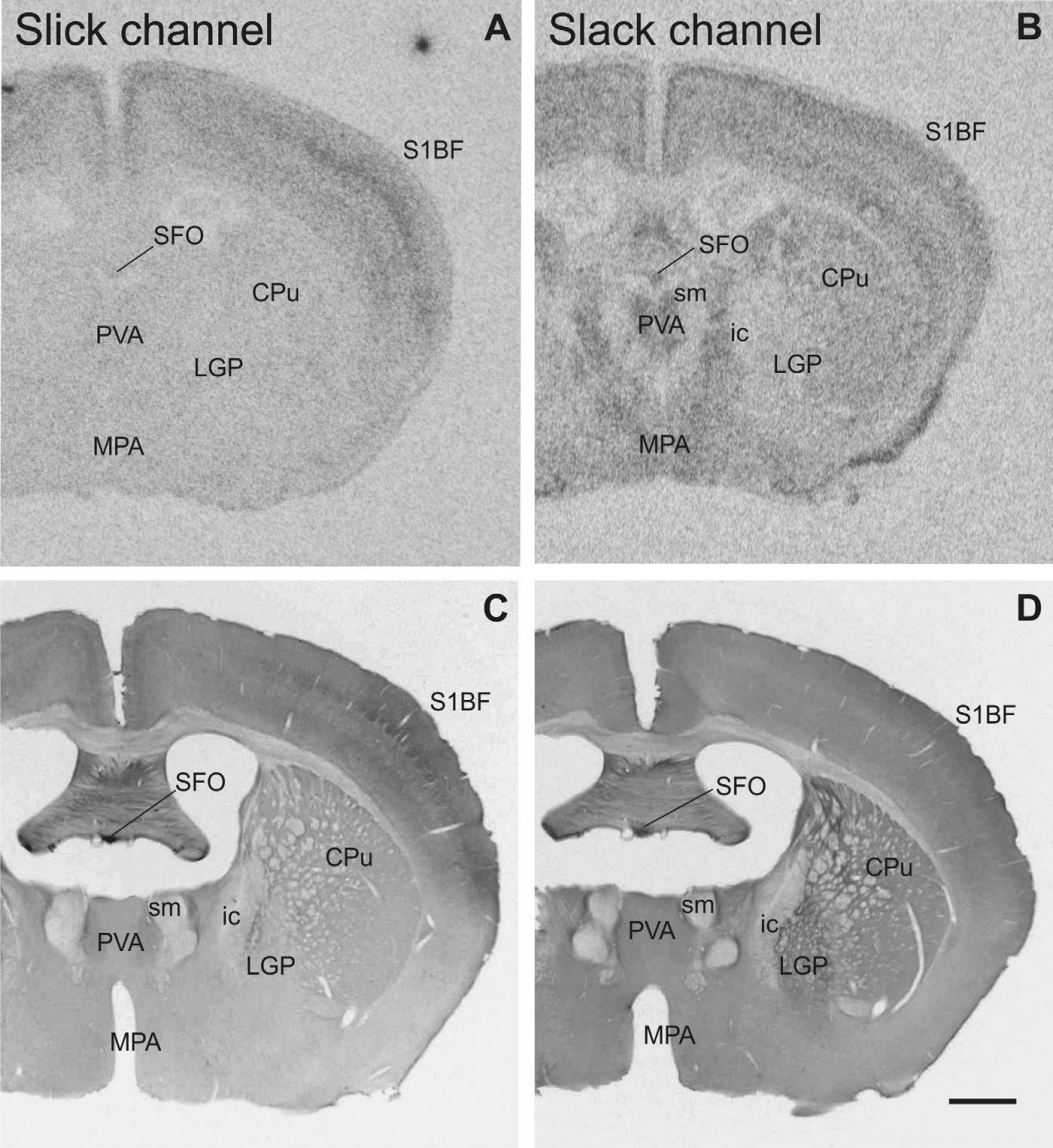
Overview of Slick and Slack channel mRNA and protein labeling in mouse brain (Bregma –0.5 mm). **A,B:** Representative autoradiographs of Slick and Slack channel *in situ* hybridization. **C,D:** Corresponding areas of Slick and Slack channel immunolabeling. **A–D:** Particularly strong *in situ* hybridization and immunohistochemical signals for Slick and Slack channel expression were detected in the subfornical organ. **D:** Slack channel immunostaining was also evident in the lateral globus pallidus. Scale bar = 1,000 μm.

**Figure 3:**
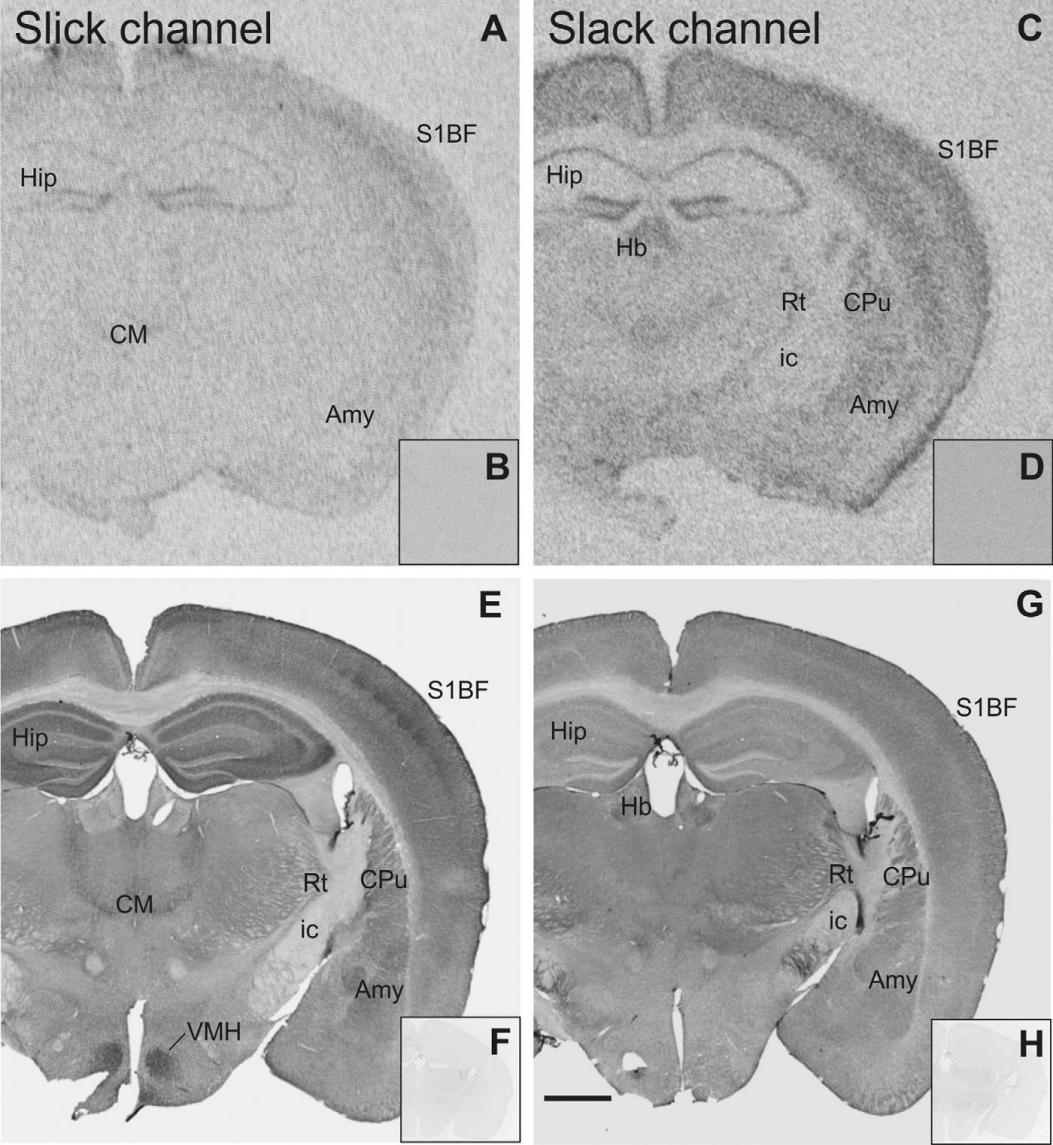
Slick and Slack channel mRNA expression and immunoreactivity in the mouse brain (Bregma approximately –1.7 mm). **A:** Strong mRNA signal for the Slick channel was detected through *in situ* hybridization in pyramidal layer of hippocampus and in granule cell layer of the dentate gyrus. **E:** Slick channel immunolabeling revealed only very weak signal in these areas. In contrast, intense Slick channel immunolabeling was observed in the strata oriens, radiatum, and lucidum as well as in the molecular layer of the dentate gyrus. Slick channel immunoreactivity was particularly strong in ventromedial hypothalamic nucleus. Moderate to strong immunolabeling for the Slick channel was detected in amygdaloid nuclei. **C:** Moderate to strong Slack channel mRNA signal in *in situ* hybridization in the amygdala and in hippocampus. **G:** Slack channel immunoreactivity was comparably low in these areas. **B,D:** Representative autoradiographs of *in situ* hybridization experiments with sense probes of Slick and Slack channel mRNA, respectively. **F:** Experiments using a nonimmune IgG of the same subtype (IgG1). **H:** Experiments in which the primary antibody was omitted served as controls for immunohistochemical experiments. Scale bar = 1,000 μm.

**Figure 4:**
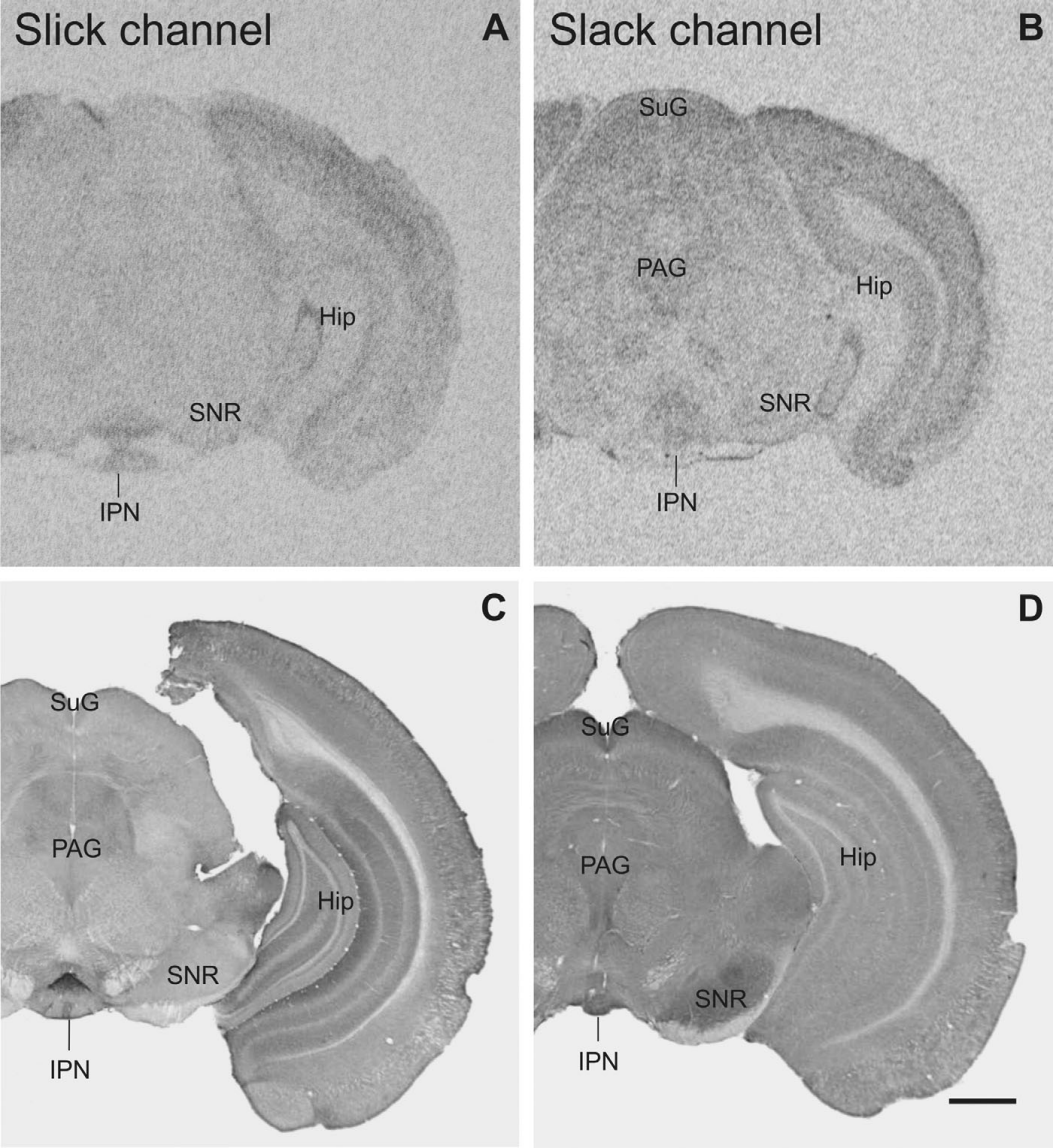
Overview of Slick and Slack channel distribution in the mouse brain (Bregma approximately –3.3 mm). **A,B:** Strong mRNA labeling was detected in *in situ* hybridization in the interpeduncular nucleus for Slick and Slack channels, respectively. **B:** Slack channel mRNA signal was moderate to strong in the superficial gray and in periaqueductal gray. **C,D:** The interpeduncular nucleus exhibited high immunoreactivity for both channels. **D:** Slack channel immunoreactivity was high in the substantia nigra pars reticulata, C whereas Slick channel immunostaining was only weak. Scale bar = 1,000 μm.

**Figure 5:**
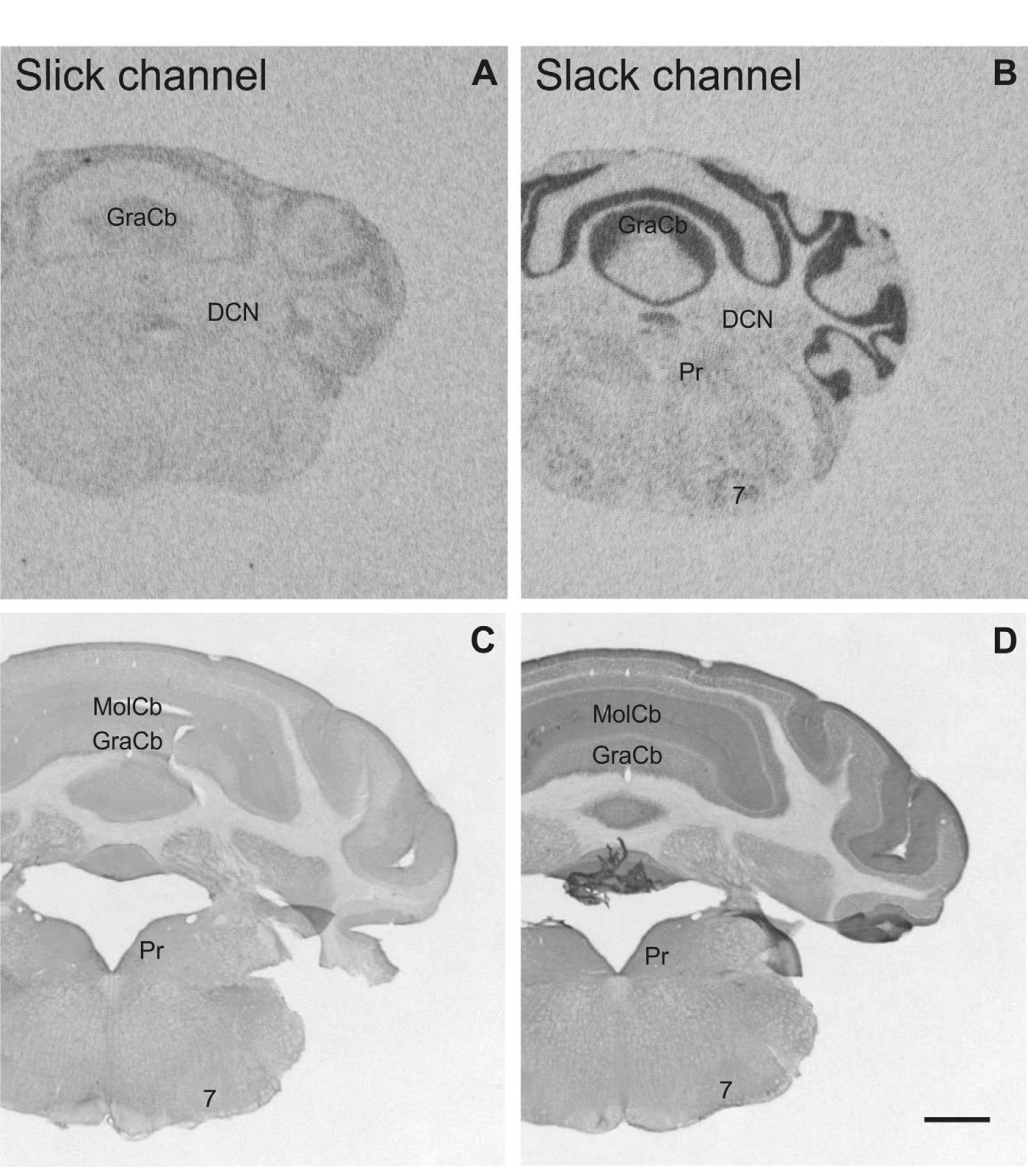
Slick and Slack channel mRNA expression and immunoreactivity in the mouse brain (Bregma approximately –6.2 mm). **A,C:** In the cerebellar cortex, Slick channel showed only low signals in *in situ* hybridization and immunohistochemical experiments. **B:** In contrast, we detected particularly strong signals for Slack channel mRNA in granule cells and in the Purkinje cell layer. **C:** Slick channel immunohistochemistry demonstrated only weak staining in cerebellar cortex. **D:** Slack channel immunostaining was moderate in the granule cell layer and only very weak staining of Purkinje cell bodies. However, strong immunolabeling was detected in the molecular layer of the cerebellar cortex. Scale bar = 1,000 μm.

Slick and Slack channels were widely distributed in the mouse brain (an overview is given in Figs. 2, 3, 4, 5; Tables 2 and 3 provide a summary of the findings). In general, *in situ* hybridization with the Slack channel‐specific probe resulted in a higher signal‐to‐noise ratio than probes targeting the Slick channel. Using four different *in situ* hybridization probes for the Slick channel, we consistently observed comparably low levels of Slick channel mRNA signal in most brain regions examined. However, both ion channel mRNAs exhibited distinct distribution patterns, with overlapping expression in certain brain regions.

**Table 2:** Slick and Slack Channel Distribution in Various Areas of the Mouse Brain^a^

	Slick (Slo2.1)		Slack (Slo2.2)	
Brain region	IHC	ISH	IHC	ISH
**Olfactory system**				
Main olfactory bulb				
Glomerular layer	+++ (d)	+++	++++ (d)	++++
External plexiform layer	+++ (d)	–/+	+++ (d)	–
Mitral cell layer	++ (d,p,v)	+/++	++ (d)	+++
Internal plexiform layer	+ (d,p)	+/++	+/++ (d)	+++
Granule cell layer	++/+++ (d,s,p)	+/++	++ (d)	+++
Accessory olfactory bulb	++/+++ (d,p)	+	+++ (d)	++
Anterior olfactory area	+++ (d,p)	+/++	+++ (d)	++++
Anterior commissure	–	–	–	–
Lateral olfactory tract	–	–	–	–
Vomeronasal nerve	–	–	–	–
**Basal ganglia and related structures**				
Nucleus accumbens	++/+++ (d,s,p,v)	n.d.	++ (d)	++/+++
Lateral globus pallidus	–/+ (d)	n.d.	+++ (d,s)	+
Medial globus pallidus	–/+ (d)	n.d.	+++ (d)	+
Ventral pallidum	–/+ (d)	n.d.	+/++ (d,s)	+/++
Claustrum	++ (d,s,v)	++	+ (d)	++
Caudate putamen	++ (d,s,p,v)	n.d.	++ (d)	++/+++
Subthalamic nucleus	–/+ (d)	n.d.	++ (d,s)	+/++
Substantia nigra pars reticulata	+ (d,s)	n.d.	++++ (d)	+
Substantia nigra pars compacta	+++ (d,s,p,v)	n.d.	++++ (d,s)	++
Nigrostriatal bundle	n.d.	n.d.	+++ (d)	n.d.
Ventral tegmental area	++ (d,p,v)	n.d.	++ (d)	+
Islands of Calleja	++++ (s,p,v)	+++	–/+ (d)	+++
Olfactory tubercle	++ (d,s,p)	+	++ (d)	++++
**Extended amygdala**				
Central amygdaloid nucleus	+++ (d,p,v)	++	+/++ (d)	++
Medial amygdaloid nuclei	++ (d,p,v)	+	–/+ (d)	++/+++
Lateral amygdaloid nucleus	+++ (d,s,p,v)	++	+/++ (d,s)	++
Basolateral amygdaloid nucleus	+++ (d,s,p,v)	++	+/++ (d,s)	++
Basomedial amygdaloid nucleus	++ (d,s,p,v)	++	–/+ (d)	++
Intercalated amygdaloid nuclei	–/+ (d)	n.d.	–	n.d.
Cortical amygdaloid nuclei	++	++	–/+	++
Substantia innominata	++/+++ (d,s,p,v)	n.d.	+ (d)	–/+
Bed nucleus stria terminalis (BST)	++ (d,p,v)	++	+	+++
Medial division BST	++ (d,p,v)	n.d.	+/++ (d)	++
Intra‐amygdaloid BST	++/+++ (d,s,p,v)	++	+ (d)	++
Stria terminalis	+ (p)	–	+ (p)	–
**Hippocampal formation**				
Subiculum	++ (d,s,p)	–/+	+ (d)	+++
Parasubiculum	+ (d)	–/+	+ (d)	+++
Presubiculum	+ (d,p)	–/+	+ (d)	+++
Fimbria hippocampi	+++ (p)	–	+++ (p)	–
CA1				
Stratum oriens	+++ (d,p)	–	+ (d,p)	–
Stratum pyramidale	–/+ (d)	+++	–	+++
Stratum radiatum	+++ (d)	–	+(d)	–
Stratum lacunosum moleculare	+ (d)	–	++ (d)	–
CA2				
Stratum oriens	+++ (d,p)	–	+ (d,p)	–
Stratum pyramidale	–/+ (d)	+++	–	+++
Stratum radiatum	++ (d)	–	+ (d)	–
CA3				
Stratum oriens	+++ (d,p)	–	++ (d,p)	–
Stratum pyramidale	–/+ (d)	++++	–	+++
Stratum lucidum	+++ (d)	–	+ (d)	–
Stratum radiatum	+++ (d)	–	+ (d)	–
Dentate gyrus				
Molecular layer	+++ (d)	–	++ (d)	–
Granule cell layer	–/+ (d)	++++	–	+++/++++
Polymorph layer	+++ (d)	–	+ (d)	–
**Septum**				
Lateral septal nucleus, dorsal	++ (d,s,p,v)	+	++ (d)	++
Lateral septal nucleus, intermediate	++++ (d,s,p,v)	n.d.	+++ (d)	+
Lateral septal nucleus, ventral	+++/++++ (d,s,p,v)	+++	++ (d)	+++
Septohippocampal nucleus	++ (d,v)	n.d.	+/++ (d)	+
Septofimbrial nucleus	++ (d,s,p)	–	++ (d)	–
Medial septal nucleus	–/+ (p,v)	n.d.	++ (d)	+
Diagonal band of Broca	–/+ (d,p,v)	n.d.	–/+ (d)	+
**Thalamus**				
Anterodorsal thalamic nucleus	++ (d)	n.d.	++ (d)	+++
Anteromedial thalamic nucleus	++ (d)	n.d.	++ (d)	+/++
Anteroventral thalamic nucleus	++ (d,p,v)	n.d.	++ (d)	+++
Ventromedial thalamic nucleus	++ (d,p,v)	n.d.	–	+
Ventral thalamic nuclei	++ (d)	n.d.	++ (d)	+
Mediodorsal thalamic nucleus	+/++ (d)	n.d.	+ (d)	++
Laterodorsal thalamic nucleus	+ (d,p)	n.d.	++ (d)	+/++
Interanterior thalamic nuclei	++ (d,s,p,v)	n.d.	–	+
Intermediodorsal nucleus	+++ (d,p)	+/++	+ (d)	++
Central lateral nucleus	++/+++ (s)	++	–/+ (d)	++
Paracentral thalamic nucleus	++/+++ (s)	++	–/+ (d)	++
Central lateral thalamic nucleus	++/+++ (s)	++	–/+ (d)	++
Lateral posterior thalamic nucleus	+/++	n.d.	+	+
Posteromedian nucleus	+++ (d,p)	n.d.	+ (d)	n.d
Posterior thalamic group	+/++ (d)	n.d.	+/++ (d)	+
Parafascicular thalamic nucleus	++ (d,p,v)	n.d.	++ (d)	++
Dorsal lateral geniculate nucleus	++ (d)	n.d.	++/+++ (d)	+
Ventrolateral geniculate nucleus magnocellular part	++ (d)	n.d.	+++ (d)	++/+++
Ventrolateral geniculate nucleus parvocellular part	++ (d)	n.d.	+ (d)	–/+
Subthalamic nucleus	–/+ (d)	n.d.	++ (d,s)	++
Parataenial nucleus	++/+++ (d,p)	n.d.	++ (d)	++
Paraventricular thalamic nucleus	++ (d,p)	n.d.	++ (d)	++/+++
Reuniens thalamic nucleus	+ (d)	n.d.	–	++
Rhomboid nucleus	+ (d)	n.d.	–	n.d.
Zona incerta	+/++ (d)	n.d.	+/++ (d)	++
Nucleus fields of Forel	++ (d,s)	n.d.	++ (d,s)	++
Submedius nucleus	++ (d)	n.d.	+ (d)	–/+
Reticular thalamic nucleus	++ (d)	n.d.	++ (d)	++/+++
Ethmoid thalamic nucleus	+ (d)	n.d.	+ (d)	n.d.
Prerubral field	+ (d)	n.d.	+ (d)	++
Pretectal nuclei	+ (d)	n.d.	+ (d)	++
Intramedullary thalamic area	+/++ (d)	n.d.	+ (d)	n.d.
Rostral interstitial nucleus of mlf	+ (d)	n.d.	++ (d)	n.d.
Medial habenular nucleus	+ (d,s,p)	n.d.	+/++ (d)	++
Lateral habenular nucleus	+/++ (d,p)	n.d.	++ (d)	++
Fasciculus retroflexus	–	–	–	–
**Hypothalamus**				
Anterior hypothalamic nucleus	++/+++ (d,s,p,v)	n.d.	+/++ (d)	++/+++
Ventromedial hypothalamic nucleus	++++ (p,v)	+	+/++ (d)	+++
Dorsomedial hypothalamic nucleus	++ (d,p,v)	n.d.	+/++ (d)	+/++
Lateroanterior hypothalamic nucleus	++ (d,p)	n.d.	+/++ (d)	+/++
Arcuate nucleus	++++ (p,v)	n.d.	++ (d)	+++
Anterior hypothalamic area	++/+++ (d,s,p,v)	n.d.	+/++ (d)	+/++
Lateral hypothalamic area	++ (d,s,p)	n.d.	+ (d)	+/++
Posterior hypothalamic area	++ (d,p,v)	n.d.	++ (d)	+++
Parastriatal nucleus	++ (d,p,v)	n.d.	–	n.d.
Periventricular nucleus	+++ (d,s,p,v)	n.d.	++ (d)	++/+++
Paraventricular nucleus	++/+++ (p)	n.d.	+/++ (d)	+++
Medial preoptic area	+ (d)	n.d.	+ (d)	++/+++
Ventromedial preoptic area	+++ (d,p,v)	n.d.	n.d.	n.d.
Lateral preoptic area	+ (d,p,v)	n.d.	–/+ (d)	++/+++
Anterodorsal preoptic nucleus	+ (d,p,v)	n.d.	–	++/+++
Median preoptic nucleus	–/+ (d)	n.d.	+/++ (d)	++/+++
Medial preoptic nuclei	++/+++ (p,v)	n.d.	+/++ (d)	++/+++
Ventrolateral preoptic nucleus	++ (d,p)	n.d.	–	++/+++
Mammillary nuclei	–	+++	++ (d)	++/+++
Supramammillary nuclei	++ (d,p,v)	+++	–	++/+++
Stria medullaris	–	–	–	–
Medial forebrain bundle	–/+	n.d.	–/+	–
Subfornical organ	+++ (s)	n.d.	++++ (s)	++++
Fornix	–/+ (p)	–	–/+ (p)	–
**Midbrain**				
Superior colliculus				
Superficial gray	+/++ (d)	n.d.	+++ (d)	++/+++
Optic nerve layer	+ (d)	n.d.	–	++
Intermediate gray layer	+/++ (d,p)	n.d.	–	++
Intermediate white layer	+ (d)	n.d.	–	–
Deep gray layer	+/++ (d,p)	n.d.	–	++
Deep white layer	+ (d)	n.d.	–	–
Inferior colliculus	–/+ (d)	n.d.	++ (d)	++
Deep mesencephalic nucleus	+ (d,p)	n.d.	++ (d)	+
Periaqueductal gray	++ (d,p,v)	n.d.	++ (d)	++
Mesencephalic trigeminal nucleus	–	n.d.	+++ (s)	n.d.
Oculomotor nucleus	++/+++ (d,s)	n.d.		
Red nucleus, magnocellular	++/+++ (d,s,p)	n.d.	++/+++ (d,s,p)	++/+++
Edinger‐Westphal nucleus	++ (d,p,v)	n.d.	++/+++ (d)	n.d.
Retrorubral field	++/+++ (d,p,v)	n.d.	++ (d,s)	n.d.
Anterior tegmental nucleus	++ (d,p,v)	n.d.	++/+++ (d)	n.d.
Paranigral nucleus	+/++ (d,p)	n.d.	++ (d)	n.d.
Interpeduncular nucleus	++++ (d,s,p,v)	++	+++ (d)	++/+++
Rhabdoid nucleus	++++ (s,p)	n.d.	++ (d)	n.d.
**Cerebellum**				
Cerebellar cortex				
Molecular layer	+/++ (d)	n.d.	+++ (d)	–
Purkinje cell layer	++ (s)	n.d.	–	++++
Granule cell layer	+ (s)	+	+++ (s)	++++
White matter	–	n.d.	–	–
Deep cerebellar nuclei				
Nucleus interpositus	+/++ (d,s,p)	n.d.	++ (d,s)	++
Medial (fastigial) nucleus	++ (d,s,p)	n.d.	++ (d,s)	++
Lateral (dentate) nucleus	+/++ (d,s,p)	n.d.	++ (d,s)	++
**Pons and medulla oblongata**				
Pontine nuclei	++ (d,s,p,v)	++	+++ (d,s,p)	++++
Dorsomedial pontine nucleus	++ (d,s)	n.d.	+++ (d)	++++
Pontine reticular nucleus, oral	++ (d,p,v)	n.d.	+/++ (d,s)	++
Pontine reticular nucleus, ventral	++ (p,v)	n.d.	++ (d)	n.d.
Pontine reticular nucleus, caudal	+ (d,s,p)	n.d.	++ (d,s,p)	+/++
Nucleus of the trapezoid body	++/+++ (s,v)	n.d.	+++/++++ (s,p)	+++
Inferior olivary complex	++/+++ (d)	n.d.	++++ (d)	++
Lateral superior olive	+/++ (d,v)	n.d.	++/+++ (d)	+/++
Superior paraolivary nucleus	+/++ (d,s)	n.d.	++/+++ (d,s,p)	+/++
Rostral periolivary region	+/++ (d,s,v)	n.d.	++/+++ (d,s)	++
Dorsal periolivary region	+/++ (d,s,p,v)	n.d.	++ (d,s)	+/++
Periolivary nuclei	+ (d,p,v)	n.d.	++ (d)	+/++
Nucleus paralemniscus	+ (p)	n.d.	+/++ (d,s)	n.d.
Lateral lemniscus nucleus	++ (d,s,p,v)	n.d.	++ (d,s)	–
Dorsal raphe nucleus	++/+++ (d,p,v)	n.d.	++ (d)	++
Dorsal raphe nucleus, caudal	++ (d,p)	n.d.	+ (d)	+/++
Dorsal raphe nucleus, inferior	++++ (p,v)	n.d.	++ (d,s)	+/++
Median raphe nucleus	++ (d,s,p)	n.d.	+++ (d)	+/++
Paramedian raphe nucleus	++ (d,s,p)	n.d.	++ (d)	+/++
Rostral linear raphe nucleus	++ (d,p,v)	n.d.	++/+++ (d)	+/++
Caudal linear raphe nucleus	++/+++ (d,p,v)	n.d.	++ (d)	++
Nucleus raphe magnus	+++ (d,p)	n.d.	++/+++ (d)	+/++
Ventrolateral tegmental area	++ (d,s,p)	n.d.	++ (d,s)	++
Dorsal tegmental nucleus	++ (d)	n.d.	++/+++ (d)	++
Subcoeruleus	++ (d,p,v)	n.d.	++ (d)	–
Locus coeruleus	++/+++ (d,s,p,v)	n.d.	+ (d)	++
Vestibular nuclei	++ (d,s)	n.d.	++ (d,s)	++
Ambiguus nucleus	+++ (d,p,v)	n.d.	n.d.	n.d.
Nucleus of the solitary tract	+++ (d,p,v)	n.d.	++/+++ (d,s,p)	++
Lateral parabrachial nuclei	+++ (d,p,v)	n.d.	+/++ (d)	++
Medial parabrachial nuclei	++ (d,p,v)	n.d.	+/++ (d)	+/++
Reticulotegmental nuclei pons	++/+++ (d,s,p,v)	n.d.	+++/++++ (d,s,p)	++++
Gigantocellular nucleus	+ (s,p)	n.d.	++ (d,s)	++
Dorsal paragigantocellular nucleus	+ (d,s,p,v)	n.d.	++ (d)	+

a Overall intensity of immunoreactivity and *in situ* hybridization labeling was evaluated using a qualitative scale of –, absent/background levels; +, weak; ++, moderate; +++, strong; ++++, very strong. Immunohistochemical staining was detected in cell bodies, processes, and varicosities or resulted in a diffuse staining pattern (dense punctae without visible underlying structure). d, diffuse; p, processes; IHC, immunohistochemistry; ISH, *in situ* hybridization; n.d., not determined; s, somatic; v, varicosities.

**Table 3:** Slick Channel Distribution in Selected Areas of the Isocortex and the Olfactory Cortex^a^

Area		Layer I	Layers II/III	Layer IV	Layer V	Layer VI
**Somatosensory cortex**						
Forelimb, hindlimb, and trunk region	ISH	++	++	+++/++++	++/+++	++
	IHC	+/++ (d)	–/+ (d,s)	++ (d)	+/++ (d)	+/++ (d)
Barrel field	ISH	++	++	+++/++++	++/+++	++
	IHC	++ (d)	++/+++ (d,s,p)	++++ (d)	++/+++ (d)	+/++ (d)
Secondary somatosensory cortex	ISH	++	++	+++/++++	++/+++	++
	IHC	+/++ (d)	++ (d,s)	++++ (d)	++ (d)	+/++ (d)
**Motor cortex**						
Primary and secondary motor cortex	ISH	++	++	*	++	++
	IHC	++ (d)	+/++ (d,s,p)	*	+/++ (d)	+ (d)
**Retrosplenial cortex**						
Retrosplenial cortex, granular	ISH	+	++	+	+	+
	IHC	++/+++ (d)	–/+ (s,p)	+/++ (d)	++ (d,s)	+ (d)
Retrosplenial cortex, agranular	ISH	+	++	*	+	+
	IHC	+++ (d,s)	+++ (d,s,p)	*	++ (d,s)	+ (d)
**Prefrontal cortex**	ISH	++/+++	++/+++	++/+++	++/+++	++/+++
	IHC	+ (d)	++ (d)	+++ (d)	++ (d)	++ (d)
**Piriform cortex**	ISH	–	++++	–		
	IHC	+ (d)	+ (s,p)	+/++ (d,s,p)		
Endopiriform nucleus	ISH	n.d.				
	IHC	+ (d)				

a While the distribution pattern of the Slack channel is similar throughout isocortical areas, Slick channel expression varies among different isocortical areas. Highest immunoreactivity was found in layer IV of primary and secondary somatosensory cortex. Immunohistochemical staining was detected in cell bodies and processes or resulted in a diffuse staining pattern (dense punctae without visible underlying structure). Note that some cortical areas are agranular regions (asterisk). d, diffuse; p. processes; IHC, immunohistochemistry; ISH, *in situ* hybridization; n.d., not determined; s, somatic.

Slick channel immunoreactivity was strongest in olfactory bulb, granular zones of isocortical regions, hippocampus, amygdala, lateral septal nuclei, distinct hypothalamic and midbrain nuclei, and regions of the brainstem. Slick channel immunohistochemistry showed widespread expression of the channel in different cell compartments. In addition to a diffuse staining, we detected pronounced staining of processes and varicosities as well as somatic staining. Intensity of Slack channel immunostaining was highest in olfactory bulb, lateral septal nuclei, basal ganglia, and distinct areas of the midbrain, brainstem, and cerebellar cortex. Immunohistochemical experiments with the Slack channel‐specific antibody revealed a primarily diffuse staining pattern of the channel. Immunolabeling of cell somata and processes was observed only occasionally.

### Olfactory system

**Figure 6:**
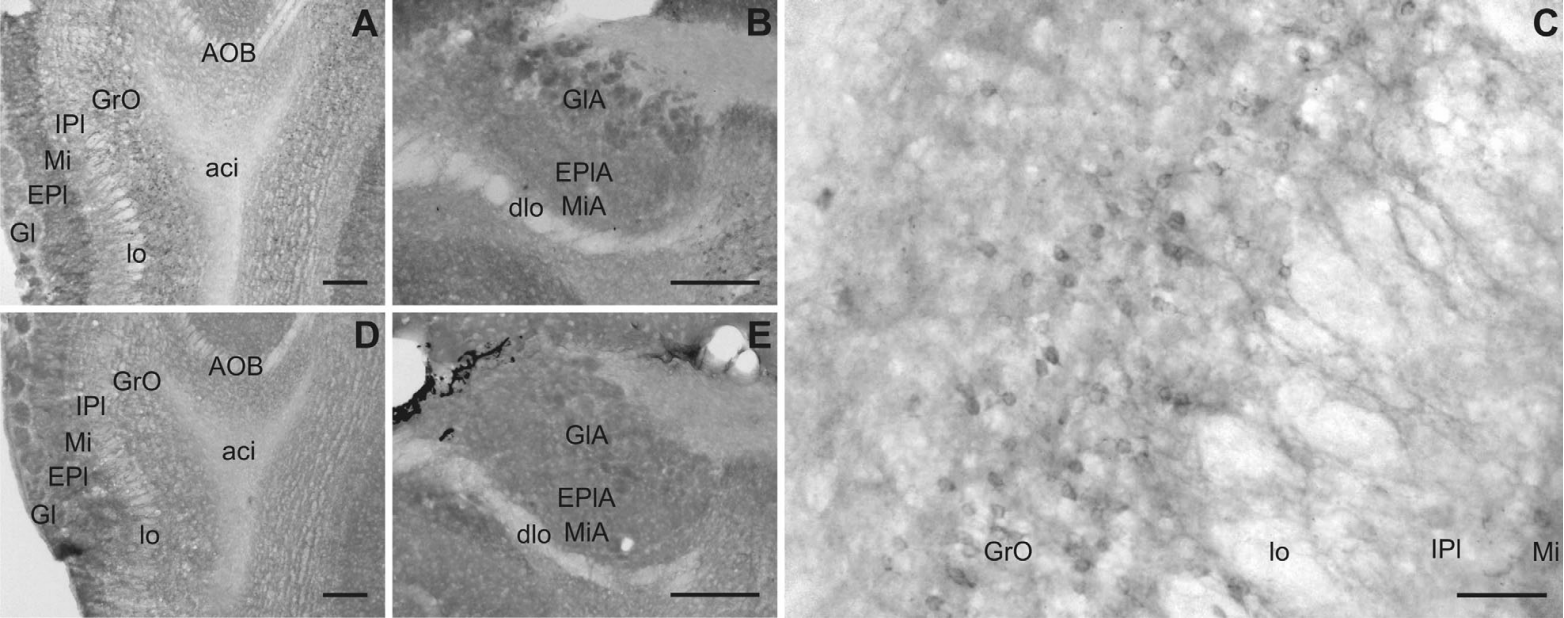
Slick and Slack channel immunoreactivity in the olfactory system. **A,D:** Slick and Slack channel immunolabeling in the main olfactory bulb, respectively (Bregma approximately 3.6 mm). **B,E:** Representative photographs of immunohistochemical experiments at the level of the accessory olfactory bulb, respectively (Bregma approximately 3.1 mm). **C:** Granule cell bodies and their dendrites were immunoreactive for Slick channels. Scale bars = 200 μm in A,B,D,E; 50 μm in C.

Both ion channels were highly expressed in the olfactory system (Fig. 6). Slick and Slack channel immunoreactivity was present in most layers of the main olfactory bulb. Strongest signals for Slick and Slack channel mRNAs were detected in periglomerular cells of the glomerular layer in the main and the accessory olfactory bulb. Strong immunoreactivity was also observed in the glomerular layer for both channels. In addition, strong Slack channel immunoreactivity was seen in the anterior olfactory area. Diffuse Slack channel immunostaining and strong *in situ* hybridization signals were detected in almost every region of the olfactory system, except for the lateral olfactory tract, vomeronasal nerve, and anterior commissure, which were devoid of Slack channel protein and mRNA signals. Despite strong immunoreactivity, the external plexiform layer did not label for Slack channel mRNA. Slick channel immunohistochemical experiments revealed strong expression in plexiform layer and in anterior olfactory nuclei. For the granule cell layer, we observed Slick channel labeling in somata and processes (Fig. 6C). The processes of granule cells extend into the mitral cell layer, where the granule cells form dendrodendritic synapses via dendritic spines with mitral/tufted cells (Mori and Yoshihara, 1995). We detected deeply stained varicosities in the mitral cell layer, possibly representing the aforementioned synapses. The mitral cell layer showed strong signals in *in situ* hybridization and immunohistochemical experiments for both channels.

### Basal ganglia and related structures

**Figure 7:**
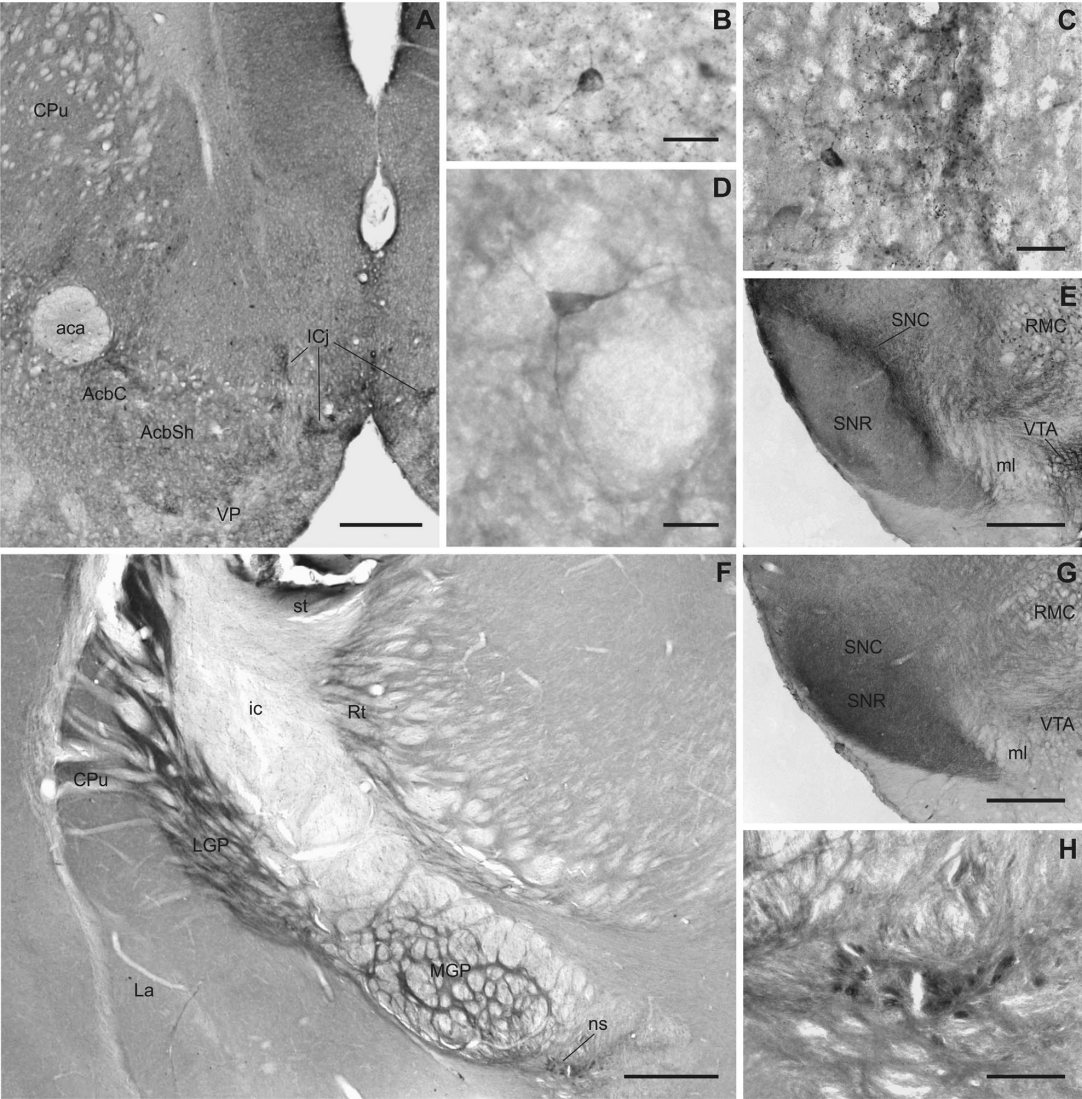
Slick and Slack channel distribution in basal ganglia and related structures. Representative photographs of Slick (**A–E**) and Slack (**F–H**) channel immunostainings of different structures of basal ganglia are shown. **A–D:** Intense Slick channel immunoreactivity was observed in the islands of Calleja and in cell bodies of the nucleus accumbens and the striatum (Bregma approximately 1.5 mm). **B:** In ventromedial parts of the striatum, small neurons with varicose processes were Slick channel immunopositive. **D:** In dorsolateral parts large neurons without varicosities were immunostained for the Slick channel. **E:** Although Slick channel immunostaining was only light in the substantia nigra pars reticulata, we observed strong staining of processes in the pars compacta. Processes in the ventral tegmental area were also deeply stained (Bregma approximately –3.5 mm). **G:** Slack channel immunoreactivity was strong in both parts of the substantia nigra, and moderate overall expression was detected in the ventral tegmental area (Bregma approximately –3.5 mm). **F:** Particularly strong Slack channel immunoreactivity was observed in the medial and lateral globus pallidus and in the nigrostriatal bundle (shown at higher magnification in **H;** Bregma approximately –1.3 mm). Scale bars = 500 μm in A,E,F,G; 25 μm in B–D; 100 μm in H.

Immunohistochemical experiments revealed a more prominent and intense staining of the Slack channel in basal ganglia compared to the Slick channel (Figs. 2C,D, 4C,D, 7). The Slack channel showed a diffuse, moderate immunostaining in almost every region of the basal ganglia; however, the strongest labeling for Slack channels was observed in lateral and medial globus pallidus as well as the substantia nigra (Figs. 4D, 7). We detected diffuse staining of the Slack channel in substantia nigra pars reticulata, and we also observed immunopositive cell bodies in the pars compacta. Particularly intense Slack channel immunoreactivity was present in the nigrostriatal bundle (Fig. 7H). In contrast, the substantia nigra pars reticulata exhibited only weak Slick channel immunoreactivity (shown in Fig. 7E). However, strong overall labeling, immunoreactive cell somata, and pronounced staining of processes and varicosities were observed in substantia nigra pars compacta (Fig. 7E). Moderate overall immunolabeling of both ion channels was detected in the ventral tegmental area. Although the Slack channel showed mainly a diffuse labeling (Fig. 7G), the Slick channel exhibited particularly intense staining of numerous processes and varicosities (Fig. 7E). Both channels showed moderate immunoreactivity in the corpus striatum. Slick channel immunostaining was seen in neuronal cell bodies, processes, and varicosities (Fig. 7A,B,D). In dorsolateral parts of the caudate‐putamen, large neurons were Slick channel immunopositive (Fig. 7D). In ventromedial parts, intense staining of processes and varicosities was observed, and neuronal cell bodies stained for the Slick channel were smaller (Fig. 7B). The islands of Calleja, a region that receives input from the substantia nigra and the ventral tegmental area, showed heavily stained cell somata, processes, and varicosities for the Slick channel (Fig. 7C) but only light immunoreactivity for the Slack channel. Despite very strong *in situ* hybridization signal in the olfactory tubercle for the Slack channel, both ion channels exhibited only moderate signals in this region in immunohistochemical experiments.

### Extended amygdala

**Figure 8:**
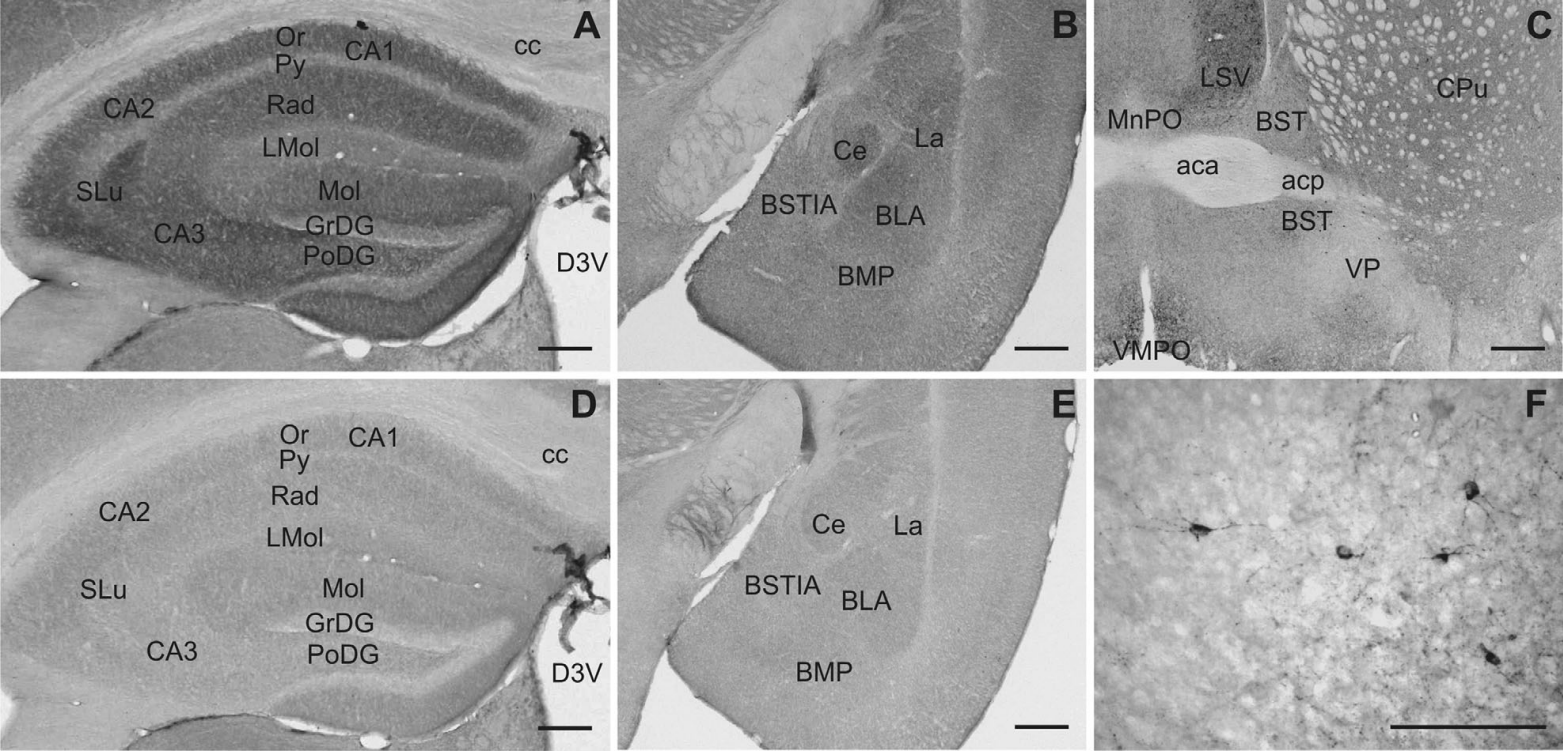
Slick and Slack channel immunolabeling in the hippocampus and extended amygdala. Although the hippocampus showed intense Slick channel immunolabeling (**A**), the Slack channel displayed comparably low immunoreactivity in this area (**D**; Bregma approximately –1.7 mm). **B:** Slick channel immunoreactivity was present in diverse nuclei of the amygdala. **E:** The Slack channel showed only moderate overall expression (Bregma approximately –1.7 mm). **C:** Overall Slick channel immunolabeling was moderate in the bed nuclei of stria terminalis (Bregma approximately 0.2 mm). **F:** At higher magnification Slick channel immunopositive neuronal cell bodies, processes, and varicosities were observed. Similar labeling was evident in all subdivisions of the bed nucleus of stria terminalis. Scale bars = 500 μm in A–E; 100 μm in F.

Slick channel immunolabeling was widely distributed in the extended amygdala, with the greatest immunoreactivity in the central, lateral, and basolateral amygdala (Figs. 3E, 8B). In addition, we observed somatic staining in lateral, basolateral, and basomedial amygdaloid nuclei, substantia innominata, and all divisions of the bed nucleus of stria terminalis (Fig. 8C,F). Deeply stained processes with varicosities were evident in almost every part of the extended amygdala, with the highest fiber density in the central and basomedial amygdala and in the bed nucleus of stria terminalis. Despite strong *in situ* hybridization signal in the bed nucleus of stria terminalis, immunohistochemical experiments revealed only light overall staining for the Slack channel in regions of the extended amygdala (Figs. 3G, 8E). However, Slack channel immunopositive cell bodies were detected in the lateral and basolateral amygdaloid nuclei.

### Hippocampal formation

We detected strong *in situ* hybridization signals for both Slick and Slack channels in pyramidal and granule cells (Fig. 3A,C). Intense Slick channel immunoreactivity was detected in strata oriens, radiatum, and lucidum of the cornu ammonis. In the dentate gyrus, Slick channel signal was strong in the molecular and polymorph layer (Figs. 3E, 8A). In contrast, immunohistochemical experiments showed only weak to moderate immunoreactivity of the Slack channel in the hippocampus proper and the dentate gyrus (Figs. 3G, 8D). Fimbria hippocampi were devoid of Slick and Slack channel mRNA signal (Fig. 3A,C); however, both showed particularly intense staining of processes in this region.

### Septum

**Figure 9:**
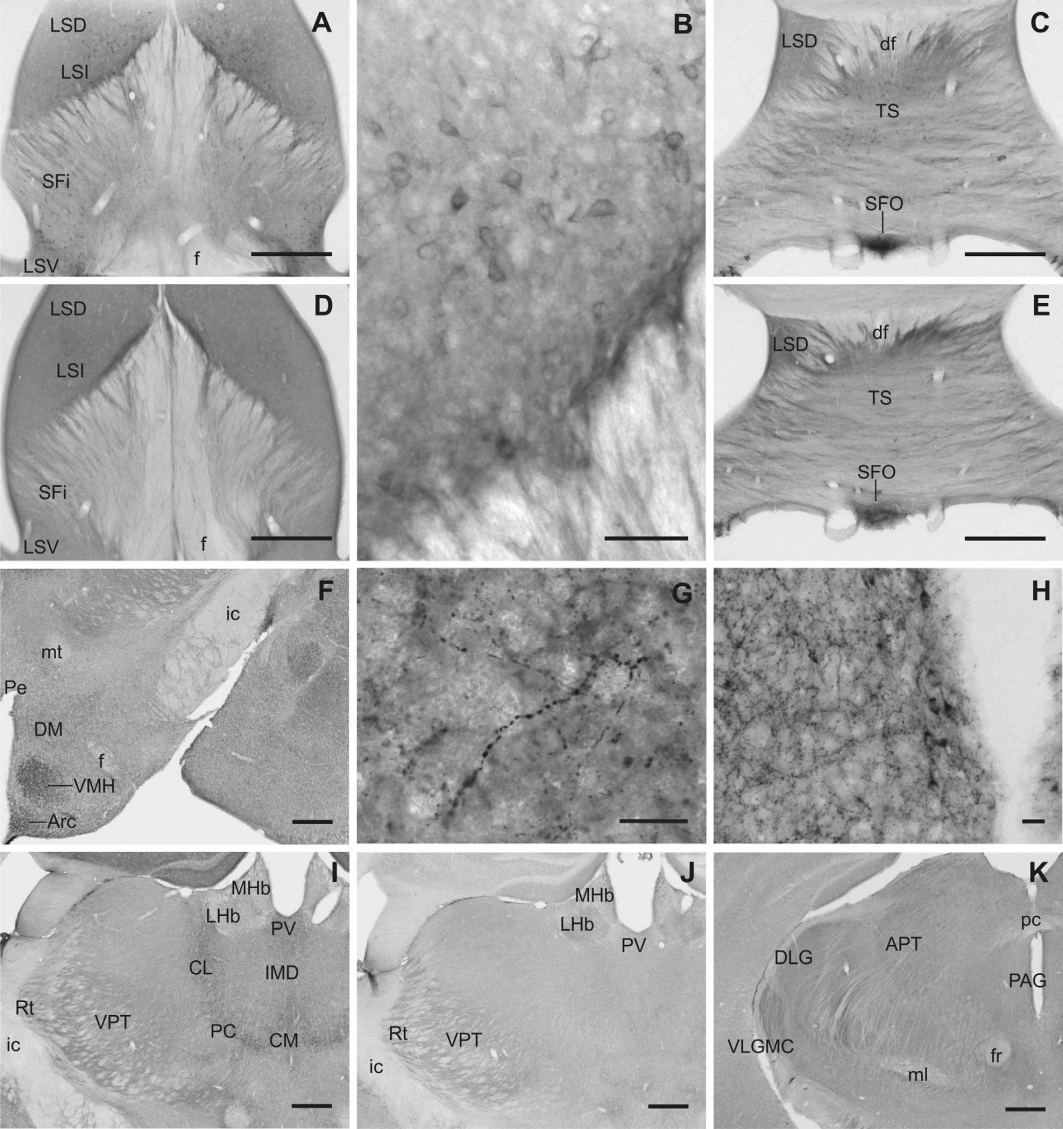
Slick and Slack channel immunoreactivity in septal nuclei, thalamus, and hypothalamic areas. **A,B:** Intense Slick channel immunolabeling of cell bodies was observed in the intermediate part of lateral septal nucleus (Bregma approximately 0.0 mm). **D:** Slack channels showed a diffuse moderate to strong immunostaining in all parts of lateral septal nuclei (Bregma approximately 0.0 mm). Slick (**C**) and Slack (**E**) channel immunolabeling showed high signals in cell bodies of the subfornical organ (Bregma approximately –4.5 mm). **F–H:** Slick channel immunoreactivity in hypothalamic areas. **F:** Prominent labeling of numerous processes and varicosities was found in the ventromedial hypothalamic and in the arcuate nucleus. In the dorsomedial hypothalamic nucleus, overall Slick channel immunoreactivity was only moderate; nevertheless, intense staining of processes and varicosities could be observed, as shown in **G** (Bregma approximately –1.6 mm). **H:** For the periventricular nucleus, we observed intense Slick channel staining of processes and varicosities. Strong staining of cell bodies and their proximal processes was observed throughout the periventricular nucleus but was most striking in ventral parts (Bregma approximately –1.6 mm). **I:** Slick channel immunoreactivity was observed in cell bodies of the centromedial, paracentral, and centrolateral thalamic nuclei. The reticular and ventral posterior thalamic nuclei showed a diffuse staining for the Slick channel (Bregma approximately –1.7 mm). **J:** Lateral habenula, reticular thalamic nucleus, and ventral posterior thalamic nuclei were immunoreactive for the Slack channel (Bregma approximately –1.7 mm). **K:** Most intense Slack channel immunoreactivity within the thalamus was observed in the magnocellular part of the ventrolateral geniculate nucleus. Moderate staining was also detected in dorsal lateral geniculate and in anterior pretectal nuclei (Bregma approximately –2.8 mm). Scale bars = 500 μm in A,C–F,I–K; 50 μm in B; 20 μm in G,H.

Within the septum, the strongest immunoreactivity for Slick and Slack channels was detected in the lateral septal nuclei (Fig. 9A,D). Immunolabeling of both channels was observed in all parts of the lateral septal nucleus, most intensely in ventral and intermediate parts. Although the Slack channel exhibited a diffuse immunohistochemical staining, Slick channel immunopositive processes and varicosities were seen in all parts of the lateral septal nuclei. The most pronounced Slick channel immunoreactivity in cell bodies was seen in the intermediate (Fig. 9B) and ventral lateral septal nucleus.

### Thalamus

In thalamic regions, Slick and Slack channels showed divergent distribution patterns. Strong Slick channel immunoreactivity was observed in the intermediodorsal and the posteromedian nuclei. In addition to a diffuse staining, we detected deeply stained processes. Slick immunopositive cell bodies were present in the central medial, paracentral, and centrolateral thalamic nuclei (Figs. 3E, 9I). A moderate diffuse staining of both channels was evident in the reticular nucleus and in the ventral posterior thalamic nuclei (Figs. 3E,G, 9I,J). Diffuse Slack channel immunolabeling was detected in the geniculate nuclei, in particular in the ventrolateral division (Fig. 9K).

### Hypothalamus

Most intense immunohistochemical staining for Slick channels was seen in the ventromedial hypothalamic nucleus and the arcuate nucleus (Figs. 3E, 9F). We observed marked staining of a notably high number of processes and varicosities in these nuclei. Strong Slick channel immunoreactivity in an intermediate number of processes and varicosities was also detected in ventromedial preoptic nucleus and periventricular nucleus. In addition, we noted very strong somatic staining in the periventricular nucleus, which was more prominent in more ventral parts of the nucleus (Fig. 9H). Highest *in situ* hybridization signal levels for Slick channels were detected in the mammillary and supramammillary nucleus. However, immunohistochemical stainings revealed only background levels in mammillary nucleus and only a moderate overall expression in supramammillary nucleus for the Slick channel. Densely packed neuronal cell bodies of the subfornical organ were heavily immunopositive for both, Slick and Slack channels (Figs. 2A–D, 9C,E). Despite strong *in situ* hybridization signal for the Slack channel in ventromedial hypothalamic nucleus, arcuate nucleus, and posterior hypothalamic area, immunohistochemical experiments revealed only weak to moderate staining for the channel in most hypothalamic areas.

### Midbrain

**Figure 10:**
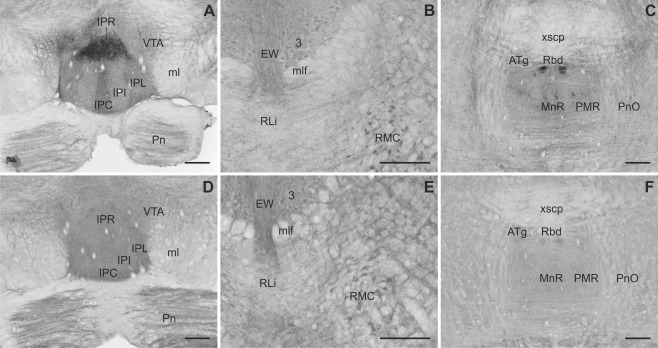
Slick and Slack channel distribution in midbrain areas. **A:** Intense Slick channel labeling was observed in neuronal cell bodies of the rostral part of the interpeduncular nucleus. Other subnuclei showed moderate to strong diffuse immunostaining. In the ventral tegmental area, mainly processes and varicosities were Slick channel immunoreactive (Bregma approximately –3.8 mm). **B:** Slick channel immunostaining was observed in cell bodies of the oculomotor and red nucleus, whereas labeling in the Edinger‐Westphal nucleus was mainly diffuse. **C:** Pronounced Slick channel immunoreactivity was evident in densely packed cell bodies of the rhabdoid nucleus (Bregma approximately –4.5 mm). **D:** Strong Slack channel staining was observed in all subnuclei of the interpeduncular nucleus (Bregma approximately –3.8 mm). Moderate to strong diffuse immunostaining was evident in the ventral tegmental area and the pontine nuclei. **E:** Slack channel staining coincided with Slick channel immunoreactivity in the oculomotor, magnocellular part of the red, and Edinger‐Westphal nuclei. **F:** The rhabdoid and anterior tegmental nuclei were moderately stained for the Slack channel (Bregma approximately –4.5 mm). Scale bars = 200 μm.

Slick and Slack channel immunoreactivity was observed in various areas of the midbrain. The interpeduncular nucleus was highly immunoreactive for the Slick channel (Figs. 4C, 10A). The Slick channel exhibited a distinct distribution pattern in the different subnuclei, with the strongest signal in the rostral subnucleus, where densely packed somata, processes, and varicosities were deeply stained (Fig. 10A). In contrast, Slack channel immunohistochemistry showed a strong diffuse staining in all subnuclei of the interpeduncular nucleus (Figs. 4D, 10D). Somata of mesencephalic trigeminal nucleus were immunopositive for the Slack channel but not for the Slick channel. Immunoreactivity of both channels was evident at moderate to strong levels in cell bodies of the red and oculomotor nuclei. A diffuse staining of both channels was evident in Edinger‐Westphal nucleus (Fig. 10B,E). Densely packed neuronal cell bodies of the rhabdoid nucleus were highly immunoreactive for Slick channels (Fig. 10C). Slack channel immunoreactivity was only moderate in this area (Fig. 10C,F).

### Isocortex and olfactory cortex

**Figure 11:**
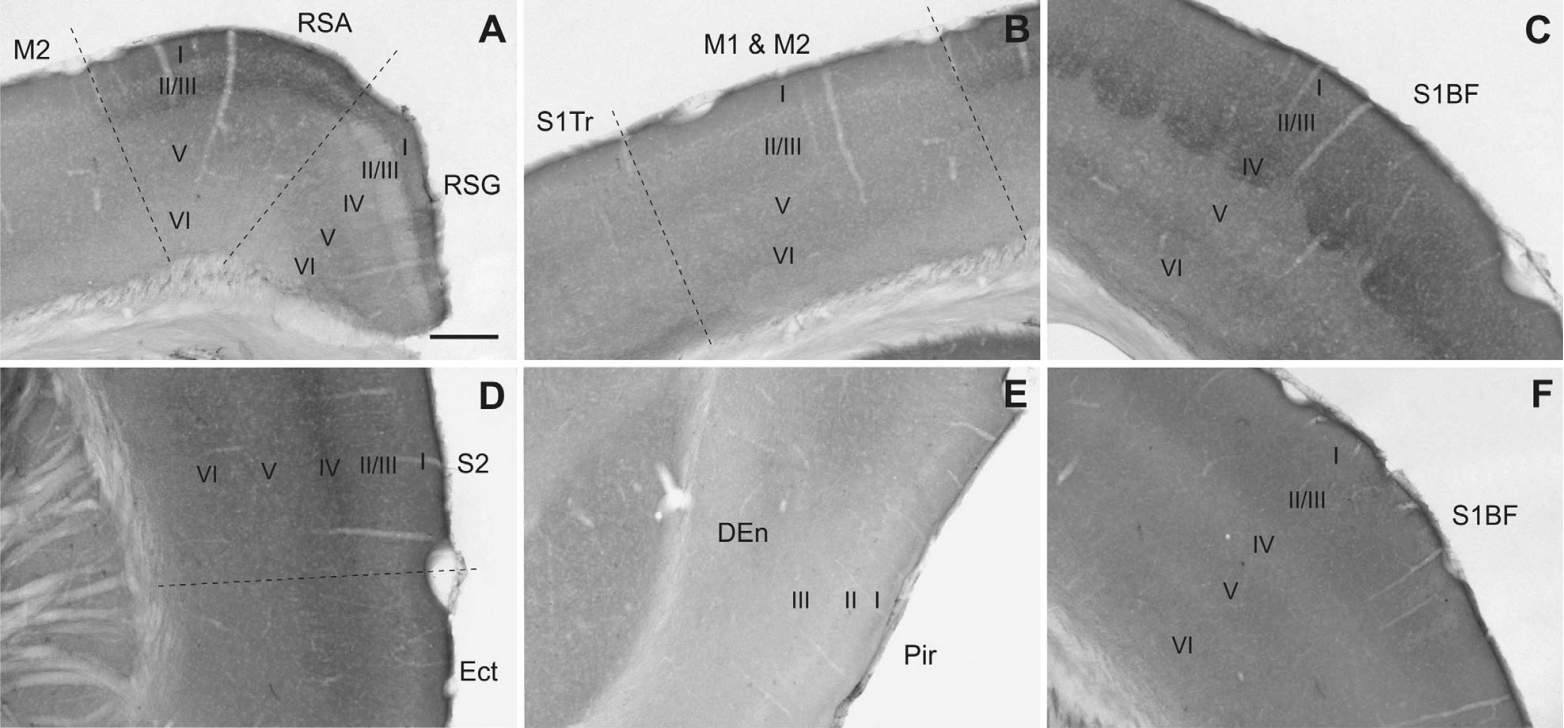
Slick and Slack channel immunostaining in cerebral cortex. **A–E:** Slick channel immunohistochemical experiments showed distinct staining patterns among different regions of the cerebral cortex. **A:** Particular intense immunoreactivity was observed in layers II/III of the agranular zone of the retrosplenial cortex and in layer IV of the primary and secondary somatosensory cortex (C,D). **C:** Note that in the barrel field of the primary somatosensory cortex the typical “cloudy” structure in layer IV is visible. In motor cortex (**B**) and piriform cortex (**E**), Slick channel immunoreactivity was comparably weak. **F:** Representative Slack channel immunostaining in the barrel field of the cerebral cortex. Slack channel immunoreactivity was not different among cortical regions. Bregma approximately –1.5 mm. Scale bar = 200 μm.

Slick channel expression pattern varied between different isocortical areas (for a summary see Table 3): In the granular retrosplenial cortex, *in situ* hybridization data showed weak mRNA expression in layers I, V, and VI. Moderate mRNA signal was detected in layers II/III (Fig. 3A). Layer I of the granular segment of the retrosplenial cortex exhibited moderate to strong diffuse immunoreactivity (Figs. 3E, 11A). Neuronal cell bodies and their proximal processes of layers II/III were immunopositive. Layers IV, V, and VI showed a mainly diffuse and weak to moderate staining. Somatic staining was evident in layer V. For the agranular retrosplenial cortex, we detected strong overall staining in layers I and II/III. In addition, in layers II/III, staining of somata and processes was observed. Layers V and VI exhibited the same staining pattern as in the granular retrosplenial cortex (Fig. 11A).

Moderate levels of Slick channel mRNA expression were detected in all layers of the primary and secondary motor cortex (Fig. 11B). Slick channel immunostaining showed a weak to moderate diffuse signal of the channel in all layers (Fig. 11B). For layers II/III, we observed immunostaining of distinct neuronal cell bodies and their proximal processes.

In the primary somatosensory cortex, both primary and secondary somatosensory cortical regions showed the highest *in situ* hybridization and immunohistochemical signals of the Slick channel in layer IV (Figs. 2C, 3E, 11C,D). A diffuse immunostaining was present in all layers, but for layers II/III we detected additional immunostaining in cell bodies and processes. In the barrel field, the typical “cloudy” structure of the thick, condensed granular cell layer was clearly distinguishable (Fig. 11C).

The outer molecular layer (layer I) and the pyramidal cell layer (layer III) of the endopiriform cortex were devoid of Slick channel *in situ* hybridization signals, whereas the outer polymorphic layer (layer II) showed a particularly strong signal. Immunohistochemical experiments showed only weak to moderate immunolabeling in all layers of the piriform cortex. Immunopositive cell somata and processes were observed in layers II and III. The endopiriform nucleus showed only light diffuse immunostaining (Fig. 11E).

We detected uniform Slick channel mRNA signals in different areas of the prefrontal cortex in all layers. Moderate to strong signals in *in situ* hybridization experiments were detected in all layers, and diffuse immunolabeling was evident in all layers, with the strongest staining in layer IV.

Moderate Slack channel immunoreactivity was present in all layers of the isocortex, except in layer IV, where immunostaining was weak (Figs. 3G, 11F). The immunostaining pattern was mainly diffuse, but through all layers we observed distinct neuronal immunopositive cell bodies. The overall immunoreactivity in the prefrontal cortex was higher than in the neocortex. *In situ* hybridization revealed that layers I and IV were devoid of Slack channel‐specific signal, but we detected moderate to strong Slack channel mRNA signal in other layers of the isocortex. Compared to layers V and VI, consistently slightly higher levels of Slack channel mRNA signal in layers II/III were detected in all cortical areas examined (Fig. 11F).

### Cerebellum

**Figure 12:**
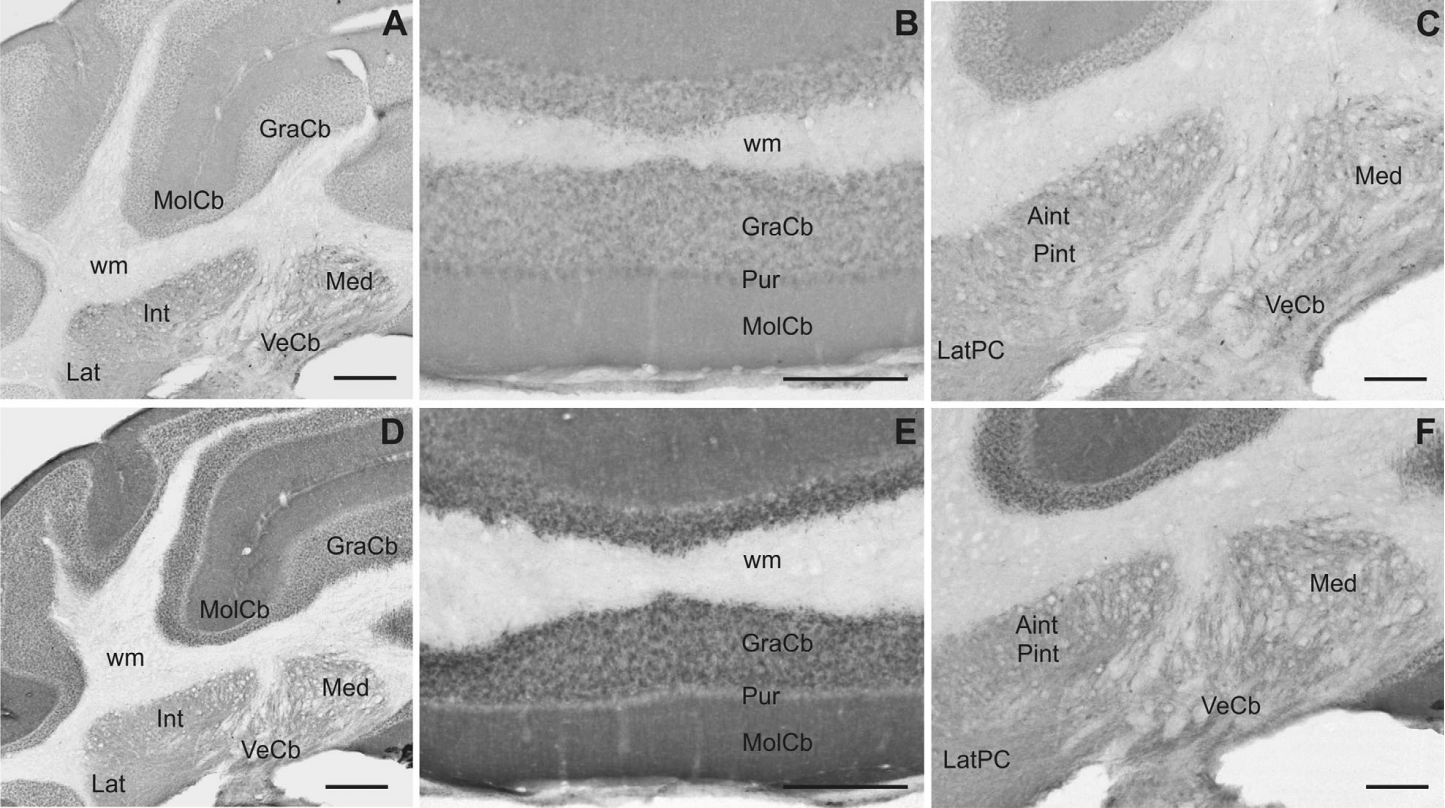
Slick and Slack channel immunoreactivity in the cerebellum. Overview of cerebellar cortex and deep cerebellar nuclei immunostained for Slick (**A**) and Slack (**D**) channels (same level in all photomicrographs, Bregma approximately –6.1 mm). Immunohistochemical staining with the Slack channel‐specific antibody showed more intense staining in the cerebellar cortex compared with the Slick channel immunostaining. **B:** Within the cerebellar cortex, the strongest Slick channel immunoreactivity was seen in the Purkinje cell layer. **E:** Intense Slack channel immunolabeling was observed in the molecular and granule cell layers of the cerebellar cortex, whereas the cell bodies of Purkinje cells were devoid of detectable signal. Slick (**C**) and Slack (**F**) channel immunoreactivity in the deep cerebellar nuclei. Neuronal cell bodies of deep cerebellar nuclei showed moderate staining for both ion channels. Scale bars = 500 μm in A,D; 200 μm in B,C,E,F.

Moderate diffuse and somatic immunoreactivity of both ion channels was seen in all deep cerebellar nuclei (Figs. 5C,D, 12A,C,D,F). In the cerebellar cortex, Slack channel mRNA exhibited notably high signals in the granule and Purkinje cell layers, whereas the molecular layer did not show detectable *in situ* hybridization signal (Fig. 5B). Strong Slack channel immunostaining was observed in the molecular and granule cell layers. In the latter, mainly the cell somata were immunopositive. However, cell bodies of Purkinje cells were devoid of Slack channel immunoreactivity (Fig. 12D,E). In contrast, Slick channel mRNA expression in the cerebellar cortex was comparably weak. Moreover, we detected only weak to moderate immunostaining in all layers, with strongest immunoreactivity in the cell bodies of Purkinje cells (Fig. 12A,B).

### Pons and medulla oblongata

**Figure 13:**
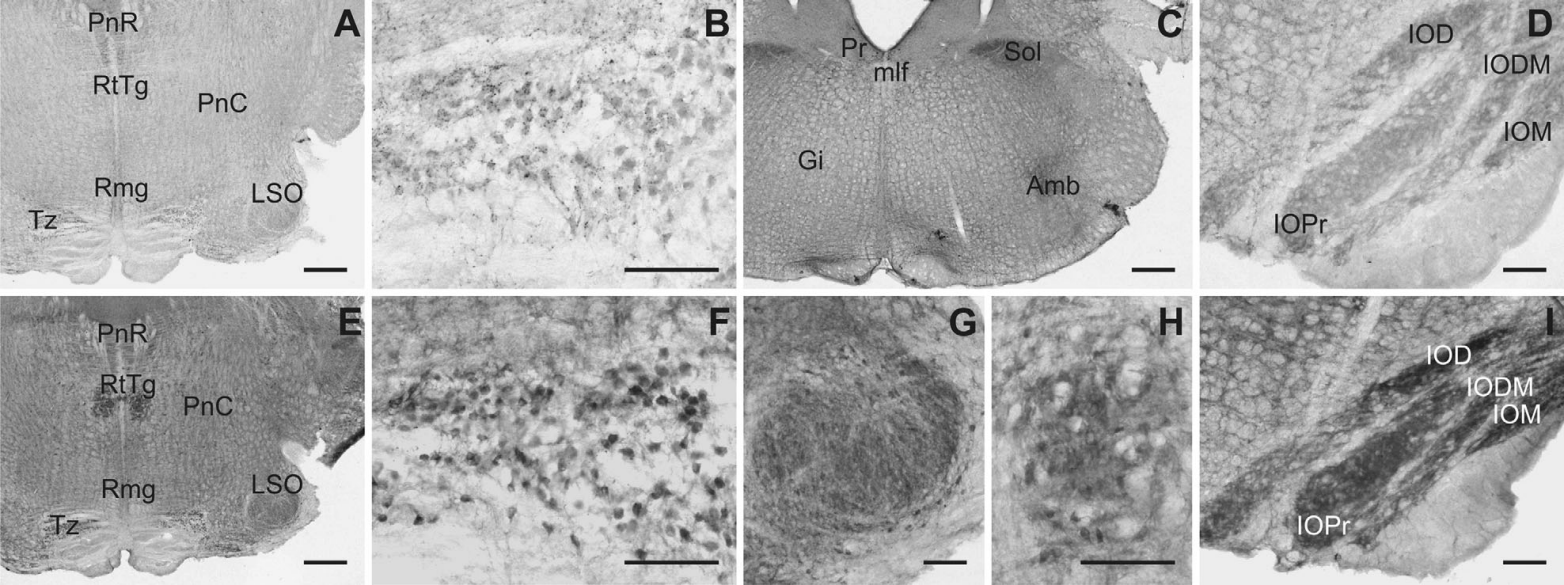
Slick and Slack channel localization in pons and medulla oblongata. Immunohistochemical experiments suggested widespread distribution of Slick (**A–D**) and Slack (**E–I**) channels in pons and medulla oblongata. Overview of Slick (**A**) and Slack (**E**) channel immunoreactivity in the brainstem (Bregma approximately –5.2 mm). Pronounced staining of cell bodies and their proximal processes was observed in the trapezoid body for Slick (**B**) and Slack (**F**) channels. **C:** Strong Slick channel immunoreactivity was detected in the nucleus of the solitary tract and the nucleus ambiguus (Bregma approximately –6.6 mm). We detected intense diffuse staining in the inferior olive for Slick (**D**) and Slack (**I**) channels (Bregma approximately –7.1 mm). Slack channel immunoreactivity was also intense in lateral superior olive (**G**) and reticulotegmental nucleus pons (**H**). For both nuclei, we observed diffuse and somatic immunostaining (Bregma approximately –5.2 mm). Scale bars = 500 μm in A,C,E; 100 μm in B,D,F–I.

Both, Slick and Slack channels were widely distributed in pons and medulla oblongata (Fig. 13). Slick channel immunoreactivity was detected throughout the raphe nuclei, and the highest expression levels were detected in the nucleus raphe magnus and the inferior dorsal raphe nucleus. The latter showed numerous heavily stained processes and varicosities. Strongly Slick channel immunopositive processes and varicosities were also detected in the nucleus ambiguus, the lateral parabrachial nucleus, and the nucleus of the solitary tract (Fig. 13C). For the latter, we also detected Slack channel immunoreactivity. Slick channel immunostaining intensity differs along the rostrocaudal axis: more caudal parts were more intensely stained. In addition to diffuse staining, we observed staining in cell somata and processes. Strongest Slack channel immunoreactivity within the pons and medulla oblongata was detected in the inferior olivary complex, the trapezoid body, and the reticulotegmental nuclei pons (Fig. 13E,F,H,I). Although in the trapezoid body immunostaining seemed to be restricted to cell bodies and their proximal processes (Fig. 13F), the reticulotegmental nucleus showed a diffuse staining (Fig. 13H). Strong Slack channel immunostaining was detected in the pontine nuclei (Fig. 10D), dorsomedial pontine nucleus, median raphe nucleus, and parvicellular trigeminal nucleus. In these areas, immunohistochemical signals were mainly diffuse, but we also detected staining of cell bodies and processes in the pontine nuclei and the reticulotegmental nucleus of pons. Widespread expression of the Slack channel was detected in medulla oblongata, especially in more caudal parts. In line with *in situ* hybridization signals, moderately immunostained cell bodies were seen in various nuclei.

### Summary: distribution pattern of Slick and Slack channels in mouse brain

Our results indicate that Slick and Slack channels exhibit distinct distribution patterns; however, in some brain regions, their expression overlapped. In summary, a substantial overlap in their expression was observed in the olfactory bulb, subfornical organ, substantia nigra pars compacta, oculomotor and red nuclei, interpeduncular and rhabdoid nuclei, nucleus of the trapezoid body, reticulotegmental nucleus of pons, and the inferior olivary complex. We also identified several brain structures in which Slick and Slack channel expression did not overlap. Slick channel immunoreactivity was seen in islands of Calleja, nuclei of the extended amygdala, hippocampal formation, and ventromedial hypothalamic and arcuate nuclei. In contrast, pronounced Slack channel immunolabeling was present in globus pallidus, substantia nigra pars reticulata and nigrostriatal bundle, and mesencephalic and parvicellular trigeminal nuclei and in parts of the cerebellar cortex.

## DISCUSSION

The present study seeks to provide a detailed description of the distribution of Slick and Slack channels in the mouse brain. This was achieved through *in situ* hybridization and immunohistochemistry. Because of current lack of Slick and Slack channel knockout animals, we used sense probes as specificity controls for Slick and Slack channel probes in *in situ* hybridization (Fig. 3B,D). Specificity of antibodies was assessed through Western blot analysis with HEK cells transfected with Slick or Slack channel encoding plasmid as well as crude plasma membranes (P2 pellet) derived from WT mouse brain. Because the paralogous Slick and Slack channels are structurally highly related (Kaczmarek, 2013), we also investigated potential cross‐reactivity of the antibodies in Western blots using transfected HEK cell lysates. Our data clearly demonstrate that the Slick channel‐specific antibody does not detect Slack channel protein and vice versa, indicating that both antibodies used are specifically recognizing their target protein (Fig. 1). In Western blots with mouse brain membranes, both antibodies labeled single bands with an apparent molecular weight that corresponds well to the predicted molecular weight (Brown et al., 2008). Experiments with a nonimmune IgG antibody of an identical subtype and experiments in which the primary antibodies were omitted served as control experiments in immunohistochemistry (Fig. 3F,H). Immunhistochemical and *in situ* hybridization control experiments resulted only in faint background staining.

Notable is the fact that our data obtained from immunohistochemical experiments are strongly supported by our *in situ* hybridization data. For example, for brain areas clearly showing immunopositive cell bodies, we also detected signals in *in situ* hybridization, such as the central and paracentral thalamic nucleus, interpeduncular nucleus, subfornical organ, and red and oculomotor nuclei. For the cerebellar cortex, we detected signals for Slack channel mRNA in granule cells and in the Purkinje cell layer. Immunohistochemical experiments showed moderate somatic staining of granule cells and only very weak staining of Purkinje cell bodies. However, strong immunolabeling was detected in the molecular layer of the cerebellar cortex, suggesting that Slack channel protein may be present in processes of granule and/or Purkinje cells that are extending into the molecular layer of the cerebellar cortex. Both, Slick and Slack channel mRNAs were detected in principal cells of the hippocampus. Immunohistochemistry revealed only very faint staining of cell bodies for both channels. Again, immunolabeling was evident in the molecular layer of the dentate gyrus for both channels as well as in the hippocampal stratum oriens, radiatum, and stratum lucidum. In general, when nonsomatic immunoreactivity was observed, labeling for mRNA was detected in at least one type of cell having processes in this area. This labeling discrepancy in *in situ* hybridization and immunohistochemistry might possibly reflect Slick and Slack channels being transported from cytoplasm to neuronal processes, as has been shown for the BK (big conductance calcium‐activated potassium channel) channel, a potassium channel of the same family the as Slick and Slack channels (Kaczmarek, 2013; Knaus et al., 1996; Sausbier et al., 2006). However, we did not specifically address this issue. Since some brain regions are heavily innervated by several neuronal groups, in‐depth analysis of such areas need be addressed in focused studies. Nevertheless, our data demonstrate that the *in situ* hybridization probes as well as the antibodies used in this study are valid tools for establishing the distribution pattern of Slick and Slack channels in the mouse brain.

In summary, our results indicate that Slick and Slack channels exhibit distinct distribution patterns; however, in some brain regions, their expression overlaps (see results section, Summary: distribution pattern of Slick and Slack channels in mouse brain). The data presented here are in line with several electrophysiological studies performed in different vertebrates. The existence of native sodium‐activated potassium currents has been proved by electrophysiological means in mitral/tufted and granule cells of the rat main olfactory bulb (Budelli et al., 2009; Egan et al., 1992a, 1992b). The presence of native sodium‐dependent currents was also shown in septum (Egan et al., 1992a), medium spiny neurons (Budelli et al., 2009), and cholinergic interneurons of the striatum in rats (Berg et al., 2007). Moreover, neurons of the rat thalamic paraventricular nucleus (Zhang et al., 2010), pons (Egan et al., 1992a), locus coeruleus (Zamalloa et al., 2009), and cerebellum (Egan et al., 1992a); the ferret reticular nucleus (Kim and McCormick, 1998); the trapezoid body of the mouse (Yang et al., 2007); and layers II/III, IV, and V of different cortical areas of cat, ferret, and rat (Budelli et al., 2009; Franceschetti et al., 2003; Sanchez‐Vives et al., 2000; Schwindt et al., 1989) expressed sodium‐activated potassium currents.

Our findings are in line with the suggested expression, but a much broader distribution of Slick and Slack channels in mouse brain has to be considered. Moreover, Slick and Slack channel immunoreactivity suggested the presence of both channels in different cell compartments. In addition to staining of punctae without clearly distinguishable underlying structure (presumably synapses), we detected pronounced staining of cell bodies, processes, and varicosities. However, our data do not distinguish between active channels positioned in the cell membrane and internalized or inactive channels.

A previous publication provided the first evidence that Slick and Slack channels are capable of forming heterotetrameric channels with unique biophysical properties *in vitro* (Chen et al., 2009). Moreover, immunoprecipitation studies with a Slack channel‐specific antibody resulted in copurification of Slick channel protein on purified membranes derived from olfactory bulb and brainstem of the rat. From their findings, the authors suggested that formation of heteromeric Slick/Slack channels occurs in these brain areas (Chen et al., 2009). We observed overlapping expression of Slick and Slack channels in these areas of the mouse brain. Moreover, we detected substantial overlap in their distribution and expression in the same cell types in several other regions, e.g., in cells of the main and accessory olfactory bulb and in the subfornical organ. Nevertheless, the presence of both channels in the same cell types does not allow conclusions on their colocalization or interaction. This should be investigated in detail for each region in future experiments.

In contrast, several brain structures expressed either Slick or Slack channels, suggesting that the channels most probably exist as homomeres in these areas. Our data suggest possible formation of homomeric Slick channels, for example, in the islands of Calleja and in the extended amygdala. Homomeric Slack channels might be present, for instance, in the globus pallidus and substantia nigra pars reticulata.

The distribution of Slick and Slack channels in the mouse brain appears partially different from that of the rat brain (Bhattacharjee et al., 2002, 2005; Joiner et al., 1998). One of the most striking discrepancies we observed in the globus pallidus. Although the Slack channel protein was not detected in rat globus pallidus (Bhattacharjee et al., 2002), we observed particular high immunoreactivity of this channel in mouse brain. Moreover, in the cerebellum, strongest Slick and Slack channel immunoreactivity was detected in the deep cerebellar nuclei of rat. In contrast, we observed the highest Slack channel immunoreactivity in the molecular and granule cell layers of the cerebellar cortex and only moderate levels in cell bodies of the deep cerebellar nuclei. Slick channel immunoreactivity appeared to be comparably weak within the cerebellum, and moderate immunoreactivity was found in the cell bodies of the Purkinje cells. Furthermore, in the neocortex, prominent Slick channel immunopositive cell bodies were detected in layers II, III, and V of the rat brain (Bhattacharjee et al., 2005). In mouse brain we observed somatic staining only in layers II/III and most pronounced expression in layer IV of the somatosensory cortex. These discrepancies might be explained, at least in part, by the use of different antibodies. While we were using an antibody recognizing all Slack channel isoforms described so far (pan‐Slack channel antibody), the aforementioned rat study employed an antibody exclusively recognizing the Slack‐B isoform (Bhattacharjee et al., 2002). On the other hand, Slick channel isoforms have not been identified so far. Other possible explanations for the observed discrepancies might be the use of different fixation and staining protocols (Bhattacharjee et al., 2002, 2005). These discrepancies might also reflect a species‐specific distribution pattern of Slick and Slack channels.

The functional roles of Slick and Slack channels in diverse brain circuits are currently not well understood. The distribution pattern presented herein could provide the neurochemical basis for additional hypothesis‐driven studies on possible functional roles. Their distribution patterns suggest possible functional roles of Slick and Slack channels in specific regulatory networks. Strong expression of Slick and Slack channels was detected in various cells types of the olfactory system, including the main and accessory olfactory bulb and the olfactory cortex and connected areas such as the hippocampus, suggesting a possible functional role of these channels in olfaction (Lledo et al., 2005). Slick channel staining was particularly strong in areas involved in goal‐directed behavior, e.g., in the ventromedial hypothalamic nucleus. This nucleus plays important roles in feeding behavior (Watson et al., 2012). Both, Slick and Slack channels were also highly expressed in the subfornical organ. This brain area is involved in the control of sodium appetite regulation and drinking behavior (Watson et al., 2012). The presence of both channels in these areas raises the possibility that they might have important functions in drinking and feeding behavior in mice. Moreover, based on the Slack channel distribution pattern, one might hypothetize that this channel plays a major role in motor function; e.g., Slack channel immunoreactivity was detected in substantia nigra, globus pallidus, and cerebellar cortex. Limbic structures involved in fear and anxiety such as amygdala, bed nucleus of stria terminalis, hippocampus, and the functionally associated prefrontal cortex (Catani et al., 2013) showed significantly high immunolabling for the Slick channel. These findings possibly indicate a functional involvement of Slick channels in emotional control. Nevertheless, these issues should be addressed in detail in separate studies. However, speculations based on neuroanatomical distribution patterns of Slick and Slack channels might stimulate both electrophysiological and behavioral studies. Because both channels are widely distributed in the CNS, the use of regional or cell‐specific adult‐onset knockout animals could be of particular interest.

## CONFLICT OF INTEREST STATEMENT

The authors declare no conflicts of interest.

## ROLE OF AUTHORS

All authors had full access to all the data in the study and take responsibility for the integrity of the data and the accuracy of the data analysis. Study concept and design: SR, CS, HGK. Acquisition of data: SR. Analysis and interpretation of data: SR, CS. Drafting of the manuscript: SR. Critical revision of the manuscript: SR, CS, HGK. Obtained funding: HGK. Administrative, technical, and material support: CS, HGK. Study supervision: CS, HGK.
